# Disruption of the gut bile acid-microbiota axis precedes severe bronchopulmonary dysplasia in preterm infants

**DOI:** 10.3389/fmicb.2025.1705965

**Published:** 2025-11-24

**Authors:** Haiyue Yu, Yongjing Guo, Jialu Li, Rong Fu, Yunfeng Zhang, Wanxu Guo

**Affiliations:** 1 Department of Pediatrics, The Second Hospital of Jilin University, Changchun, China; 2 Department of Neonatology, The Second Hospital of Jilin University, Changchun, China; 3 Children's Disease Diagnosis and Treatment Center, The Second Hospital of Jilin University, Changchun, China

**Keywords:** bronchopulmonary dysplasia (BPD), preterm infants, bile acids, gut microbiota, gut-lung axis, multi-omics, machine learning, biomarkers

## Abstract

**Background:**

Bronchopulmonary dysplasia (BPD) remains a major cause of morbidity in preterm infants, yet current diagnostic criteria are delayed and underlying mechanisms are incompletely defined. Evidence suggests that intestinal dysbiosis may influence pulmonary outcomes via the gut–lung axis, but the metabolic mediators of this interaction remain unclear.

**Methods:**

We conducted a prospective cohort study of 50 preterm infants (≤ 32 weeks gestation), stratified by BPD severity at 36 weeks. Stool samples collected on postnatal day 7 underwent 16S rRNA sequencing and targeted bile acid metabolomics. Differential features were identified via multivariate statistics and LEfSe. Spearman correlation analysis explored bile acid–microbiota interactions. An interpretable machine learning model (XGBoost) incorporating bile acid and microbial features was developed and validated using five-fold cross-validation and an independent test set.

**Results:**

Infants with severe BPD showed significantly reduced levels of 16 bile acids—including primary, secondary, and sulfated species—compared to non-BPD controls. Gut microbiome β-diversity differed significantly among groups, with enrichment of opportunistic Proteobacteria (e.g., *Brevundimonas*) in severe BPD. Negative correlations were observed between depleted bile acids and enriched bacterial genera. The XGBoost model predicted BPD severity with 80% accuracy (AUC = 0.91), leveraging key features such as chenodeoxycholic acid (CDCA), hyocholic acid (HCA), and *Brevundimonas*.

**Conclusions:**

Preterm infants who develop severe BPD exhibit early disruption of the bile acid–microbiota axis, characterized by reduced bile acid levels and enrichment of opportunistic taxa. Integrating these features within interpretable machine-learning models enables accurate early risk stratification and provides mechanistic insights beyond traditional inflammation-based frameworks. Validation in larger, multicenter cohorts is warranted to refine biomarker panels and explore targeted interventions that modulate bile acid signaling or microbial ecology to prevent or attenuate BPD.

## 1 Introduction

Bronchopulmonary dysplasia (BPD) is the most common chronic respiratory complication among extremely preterm infants, characterized by impaired alveolarization (alveolar simplification) and abnormal pulmonary microvascular development. Although advances in perinatal care have improved survival, the incidence of BPD has not declined and it remains a major contributor to long-term respiratory morbidity and reduced quality of life (Thekkeveedu et al., 2017; Zhu, 2024). Current diagnostic criteria rely on oxygen requirement at 28 days of life or at 36 weeks' postmenstrual age (4), an inherently delayed assessment. The pathogenesis of BPD also remains incompletely defined: classic models emphasize antenatal inflammation, ventilator-induced injury, and oxygen toxicity leading to dysregulated injury-repair cycles (Thekkeveedu et al., 2017; Dankhara et al., 2023), but these factors alone do not capture the full disease spectrum. This combination of delayed diagnosis and incomplete mechanistic insight hampers timely intervention.

The gut-lung axis has emerged as a systemic framework linking intestinal homeostasis to pulmonary immunity and disease. Bidirectional crosstalk through immune, metabolic, and neuroendocrine pathways suggests that gut microbial balance can shape distal pulmonary immune tone and inflammatory responses (Tirone et al., 2019). Preterm infants commonly exhibit intestinal dysbiosis–marked by delayed colonization, reduced microbial diversity, and overrepresentation of opportunistic taxa such as Proteobacteria–which has been associated with higher risks of necrotizing enterocolitis (NEC) (Pammi et al., 2017), late-onset sepsis (LOS) (Mai et al., 2013), and BPD (Chen, 2021). However, the molecular mediators and downstream signaling that connect intestinal dysbiosis to lung injury remain poorly defined.

Bile acids are increasingly recognized as signaling molecules at the host-microbiota interface, acting through receptors such as Farnesoid X Receptor (FXR) and Takeda G-protein-coupled Receptor 5 (TGR5) to influence systemic immunity and organ development (Fleishman and Kumar, 2024; Godlewska et al., 2022). Preclinical data indicate that bile acid dysregulation–via toxic pulmonary accumulation or systemic deficiency that impairs anti-inflammatory signaling–may contribute to neonatal lung injury (Zecca et al., 2008; De Luca, 2022) Yet, whether bile acid imbalance contributes to BPD in preterm infants, particularly through its interaction with intestinal dysbiosis, remains unclear.

Machine learning provides a powerful means to interrogate such complex host-microbiota-metabolite systems. By integrating multi-omics datasets, these methods can capture nonlinear relationships, identify key biomarkers, and enable accurate, interpretable prediction (Li et al., 2022; Licciardi et al., 2024). To date, most BPD prediction studies have focused on clinical variables such as gestational age and birth weight (Leigh et al., 2022; Yoneda et al., 2024; Hwang et al., 2023; Choi, 2025); integrative approaches combining bile acid metabolomics with gut microbiome profiling have not been reported.

Against this background, we conducted a prospective observational cohort study to examine the contribution of early-life (postnatal day 7) bile acid profiles and gut microbial composition to BPD and to explore their interaction. We hypothesized that infants who develop severe BPD exhibit early reductions in intestinal bile acids together with dysbiosis, and that these disturbances act in concert through bile acid-microbiota crosstalk to drive disease progression. To test this, we built interpretable machine-learning models using differential metabolite and microbial features to evaluate early prediction of BPD severity. This study is novel in two respects: (i) it integrates bile acid metabolomics and gut microbiome data in preterm infants to elucidate their cross-talk in BPD pathogenesis, and (ii) it develops a bile acid-microbiota-based, interpretable machine-learning framework for very early risk prediction. These advances offer a metabolic-microbial perspective on BPD mechanisms and lay the groundwork for precision risk stratification and targeted interventions.

## 2 Materials and methods

### 2.1 Study design and ethics

This single-center, prospective observational cohort study was conducted in the neonatal unit of the Second Hospital of Jilin University. The study protocol, including predefined inclusion criteria, sampling schedule, and analysis plan, was designed and approved by the institutional ethics committee in July 2024 (Approval No.: 2024-309) before patient recruitment began.

Enrollment of eligible preterm infants commenced in November 2024 and continued until February 2025. Clinical data and biological samples were collected in real time during hospitalization according to the study protocol, and all infants were followed until discharge. Written informed consent was obtained from parents or legal guardians prior to enrollment.

### 2.2 Study participants

#### 2.2.1 Inclusion and exclusion criteria

*Inclusion criteria*: (i) preterm infants with gestational age (GA) ≤ 32 weeks, born at and admitted to the Second Hospital of Jilin University (GA determined by last menstrual period and confirmed by first-trimester ultrasound); (ii) no major congenital anomalies or genetic syndromes; and (iii) guardian consent. *Exclusion criteria*: (i) complex congenital heart disease; (ii) malformations of the central nervous, gastrointestinal, or respiratory systems; (iii) requirement for invasive respiratory support due to surgery or other non-BPD indications; (iv) other major anomalies (e.g., congenital diaphragmatic hernia, chromosomal abnormalities); (v) anticipated survival <28 days; or (vi) refusal to participate.

#### 2.2.2 Group definitions

BPD status at 36 weeks' postmenstrual age was determined according to the 2018 NIH workshop consensus (Higgins et al., 2018). Infants were classified as Non-BPD (NonBPD7), non-severe BPD (BPD7m; including mild and moderate cases), or severe BPD (BPD7s) based on oxygen requirement and respiratory support.

### 2.3 Sample collection and processing

On postnatal day 7 (PND7), ~1 g of stool was collected with a sterile disposable spatula, transferred to sterile cryovials, and immediately stored at −80°C until 16S rRNA gene sequencing and targeted bile acid metabolomics.

### 2.4 16S rRNA gene sequencing and bioinformatics

#### 2.4.1 DNA extraction

Total genomic DNA was extracted using the MagBeads FastDNA Kit for Soil (116564384, MP Biomedicals, CA, USA) according to the manufacturer's protocol and stored at −20°C until further analysis. DNA concentration and purity were assessed using a NanoDrop NC2000 spectrophotometer (Thermo Fisher Scientific, Waltham, MA, USA), and integrity was verified by agarose gel electrophoresis.

#### 2.4.2 16S rRNA gene amplicon sequencing

The V3–V4 hypervariable region of the bacterial 16S rRNA gene was amplified using primers 338F (5′-ACTCCTACGGGAGGCAGCA-3′) and 806R (5′-GGACTACHVGGGTWTCTAAT-3′) with sample-specific 7-bp barcodes. Each 20 μl PCR reaction contained 5 μl of 5 × buffer, 0.25 μl FastPfu DNA polymerase (5 U/μL), 2 μl dNTPs (2.5 mM), 1 μl of each primer (10 μM), 1 μl DNA template, and 14.75 μl ddH_2_O. The PCR program consisted of an initial denaturation at 98°C for 5 min, followed by 26 cycles of 98°C for 30 s, 52°C for 30 s, and 72°C for 45 s, with a final extension at 72°C for 5 min. Amplicons were purified with VAHTS DNA Clean Beads (Vazyme, Nanjing, China), quantified with the Quant-iT PicoGreen dsDNA Assay Kit (Invitrogen, Carlsbad, CA, USA), pooled in equimolar amounts, and sequenced on the Illumina NovaSeq 6000 platform (2 × 250 bp paired-end, SP Reagent Kit, 500 cycles) at Shanghai Personal Biotechnology Co., Ltd (Shanghai, China).

### 2.5 Targeted bile acid metabolomics

#### 2.5.1 Chemicals and reagents

HPLC-grade acetonitrile (ACN) and methanol (MeOH) were obtained from Merck (Darmstadt, Germany). Milli-Q water (Millipore, Bradford, USA) was used throughout. Bile acid standards were purchased from CNW (Shanghai, China) and IsoReag (Shanghai, China). Acetic acid and ammonium acetate were obtained from Sigma-Aldrich (St. Louis, MO, USA). Stock solutions of each standard were prepared at 1 mg/ml in MeOH and stored at −20°C, then diluted with MeOH to working solutions prior to analysis.

#### 2.5.2 Sample preparation and extraction

Approximately 20 mg of sample was ground using a ball mill and extracted with 495 μl of MeOH containing 5 μl of internal standard mixture (10 μg/ml). Extracts were incubated at −20°C for 10 min to precipitate proteins, followed by centrifugation at 12,000 rpm for 10 min at 4°C. The supernatant was passed through a protein precipitation plate prior to LC–MS analysis.

#### 2.5.3 HPLC conditions

Chromatographic separation was performed on an LC-ESI-MS/MS system (UHPLC, ExionLC^TM^ AD; MS, SCIEX 6500 Triple Quadrupole). The column was a Waters ACQUITY UPLC HSS T3 C18 (100 mm × 2.1 mm, 1.8 μm). Mobile phases were (A) water with 0.01% acetic acid and 5 mmol/L ammonium acetate, and (B) acetonitrile with 0.01% acetic acid. A linear gradient was applied: 5–40% B (0–1 min), 40–50% B (1–6 min), 50–75% B (6–11 min), 75–95% B (11–13 min), held at 95% B (13–15 min), and re-equilibrated to 5% B (16–17.5 min). Flow rate: 0.35 ml/min; column temperature: 40°C; injection volume: 3 μL.

#### 2.5.4 ESI-MS/MS conditions

Mass spectrometry was performed on a QTRAP 6500+ LC–MS/MS system (SCIEX) equipped with an ESI Turbo Ion-Spray source operating in negative ion mode under Analyst 1.6.3 control. Source parameters: source temperature 550°C, ion spray voltage −4, 500 V, curtain gas 35 psi. Bile acids were quantified using scheduled multiple reaction monitoring (MRM). Multiquant 3.0.3 (SCIEX) was used for peak integration and quantification, with declustering potential (DP) and collision energy (CE) individually optimized for each bile acid. Specific MRM transitions were monitored according to expected retention time windows.

### 2.6 Statistical analysis

Comprehensive maternal and neonatal baseline data were collected, including sex, mode of delivery, maternal infection, obstetric medication (magnesium sulfate, antenatal corticosteroids, antibiotics), pregnancy complications, plurality, gestational age, birth weight, Apgar score, and early postnatal interventions (endotracheal intubation, surfactant administration, caffeine, vasoactive agents). Categorical variables were summarized as counts (percentages) and compared using the χ^2^ test or Fisher's exact test when >20% of cells had an expected frequency <5. Continuous variables were tested for normality with the Shapiro–Wilk test. Normally distributed data were expressed as mean ± standard deviation (SD) and analyzed by one-way ANOVA, while skewed data were expressed as median (interquartile range, IQR) and compared by the Kruskal–Wallis *H*-test. All tests were two-sided with α = 0.05. Statistical analyses were performed in Python (v3.9) using the SciPy library.

Microbiome bioinformatics were performed with QIIME 2 (2024.10) with slight modification according to the official tutorials (https://docs.qiime2.org/2024). Briefly, raw sequence data were demultiplexed using the demux plugin, followed by primer trimming with the cutadapt plugin (Martin, 2011). Sequences were quality-filtered, denoised, merged, and chimera-removed using the DADA2 plugin (Callahan et al., 2016). Non-singleton amplicon sequence variants (ASVs) were aligned with mafft (Katoh et al., 2002) and used to construct a phylogeny with fasttree2 (Price et al., 2009). For downstream analyses, α-diversity indices [Chao1 (Chao, 1984), Simpson (Simpson, 1949), Shannon (Shannon, 1948), Good's coverage (Good, 1953), Observed species, and Pielou's evenness (Pielou, 1966)] were calculated, and inter-group comparisons were performed using the Kruskal–Wallis test. β-diversity was assessed using Bray–Curtis (Bray and Curtis, 1957) and UniFrac distances (Lozupone and Knight, 2005), visualized by principal coordinate analysis (PCoA). Differences in microbial community structure between groups were tested using PERMANOVA (Adonis) with 999 permutations. Taxonomic differences at the genus level were identified by linear discriminant analysis effect size (LEfSe), with LDA score > 2.0 and *P* < 0.05 considered significant.

For bile acid profiling, univariate analysis used the Kruskal–Wallis H test to compare relative concentrations across groups, with *P* < 0.05 as the selection threshold. Multivariate analysis was performed using orthogonal partial least squares discriminant analysis (OPLS–DA) to visualize inter-group separation; model validity was evaluated by 999-time permutation testing, and models with *Q*^2^>0.3 and permutation *P* < 0.05 were deemed reliable. Differential bile acids were defined through pairwise group comparisons using combined criteria: VIP > 1, *P* < 0.05, and absolute log_2_ fold change (|log_2_FC|) > 1. When different numbers of differential bile acids were obtained across group pairs, the comparison yielding the largest differential set was used as the reference for subsequent analyses.

### 2.7 Integrated analysis and machine learning

#### 2.7.1 Interaction network analysis

Spearman correlations (ρ) were computed between differential bile acids and bacterial genera; significant associations (|ρ|>0.5, *P* < 0.05) were visualized in Cytoscape v3.9.1.

#### 2.7.2 Modeling and validation

All machine learning analyses were performed in Python using scikit-learn, imbalanced-learn, and XGBoost. The modeling pipeline was standardized across experiments to ensure comparability. First, clinical and omics variables were preprocessed by removing constant features and applying *z*-score normalization. To identify informative predictors, a Kruskal–Wallis test across BPD severity groups was performed for each variable, followed by Benjamini–Hochberg false discovery rate (FDR) correction; the top 20 features with the smallest adjusted *p*-values were retained as candidate inputs. To address class imbalance among BPD categories, Synthetic Minority Over-sampling Technique (SMOTE) was applied on the training data prior to model fitting. Stratified train–test partitioning was adopted (70:30 split), with the option of repeated stratified shuffling or five-fold cross-validation for robustness checks.

Multiple classifiers were benchmarked, including tree-based models [Extreme Gradient Boosting (XGBoost), Random Forest (RF)], linear models [Logistic Regression (LR)], kernel methods [Support Vector Machine (SVM)], instance-based learning [*k*-nearest neighbors (KNN)], and Naïve Bayes (NB). For scale-sensitive algorithms, normalization was incorporated within imbalanced-learn pipelines to prevent information leakage. Hyperparameters of XGBoost and RF were tuned toward stability (e.g., depth restriction, subsampling, regularization), while ensemble modeling was further explored by implementing a soft-voting strategy that linearly combined the class-probability outputs of XGBoost and RF, with the optimal fusion weight (α) selected on a held-out validation subset by maximizing macro-averaged F1-score.

Model evaluation was conducted on the independent test set. Primary metrics included overall accuracy, macro-averaged F1 (F1-macro), and macro-averaged one-vs-rest AUROC. For cross-validation settings, mean performance and standard deviation were reported. Robustness was further examined through repeated random splits (100 iterations) and permutation tests (1,000 label shuffles) to estimate empirical *p*-values. Model interpretability was assessed using SHapley Additive exPlanations (SHAP), where both global summary plots and local attribution waterfall plots were generated. Additionally, SHAP stability was quantified by repeating feature attribution across multiple resampled datasets and calculating the frequency of top-*k* features and their pairwise Jaccard similarity.

## 3 Results

### 3.1 Clinical characteristics

A total of 50 preterm infants were enrolled: Non-BPD (*n* = 17), non-severe BPD (*n* = 11; including one mild and 10 moderate cases), and severe BPD (*n* = 22). Baseline characteristics—including sex, mode of delivery, maternal infection, obstetric medications (magnesium sulfate, steroids, and antibiotics), pregnancy complications, early antibiotic exposure, and both the timing and type of feeding–did not differ significantly among groups (all *P*>0.05). Gestational age and birth weight decreased with increasing BPD severity (Non-BPD: 32.0 weeks, 1,760 g; Mild BPD: 29.9 weeks, 1,510 g; Severe BPD: 29.2 weeks, 1075 g; *P* < 0.05). The proportion of singletons was highest in the Mild BPD group (100%) and lowest in the Non-BPD group (58.8%) (Table 1).

**Table 1:** Baseline characteristics of the study population.

**Variable**	**Non-BPD (*n* = 17)**	**Mild-BPD (*n* = 11)**	**Severe-BPD (*n* = 22)**	***p*-value**	**Statistical test**
Gender, M (%)	12 (70.6%)	6 (54.5%)	10 (45.5%)	0.2907	χ^2^
Delivery mode, C-section (%)	15 (88.2%)	9 (81.8%)	20 (90.9%)	0.8518	Fisher
Maternal infection, *n* (%)	2 (11.8%)	3 (27.3%)	5 (22.7%)	0.5991	Fisher
Fetus number, singleton (%)	10 (58.8%)	11 (100.0%)	19 (86.4%)	0.0229^*^	Fisher
Maternal MgSO_4_, *n* (%)	10 (58.8%)	6 (54.5%)	17 (77.3%)	0.3200	χ^2^
Maternal steroid, *n* (%)	16 (94.1%)	8 (72.7%)	20 (90.9%)	0.2751	Fisher
Maternal antibiotics, *n* (%)	15 (88.2%)	11 (100.0%)	21 (95.5%)	0.5936	Fisher
Maternal complication, *n* (%)	16 (94.1%)	10 (90.9%)	21 (95.5%)	1.0000	Fisher
Intubation, *n* (%)	17 (100.0%)	10 (90.9%)	21 (95.5%)	0.6954	Fisher
Surfactant use, *n* (%)	16 (94.1%)	11 (100.0%)	22 (100.0%)	0.5618	Fisher
Caffeine use, *n* (%)	17 (100.0%)	11 (100.0%)	22 (100.0%)	1.0000	Fisher
Antibiotic exposure (≤ 7 days), *n* (%)	17 (100.0%)	11 (100.0%)	22 (100.0%)	1.0000	Fisher
Formula feeding ≤ 7 days, *n* (%)	16 (94.1%)	11 (100.0%)	22 (100.0%)	0.5618	Fisher
Feeding start day, median (IQR)	2.0 (1.0–3.0)	2.0 (1.0–4.0)	2.0 (2.0–3.0)	0.683	Kruskal–Wallis
Vasoactive drug, *n* (%)	12 (70.6%)	11 (100.0%)	15 (68.2%)	0.1088	Fisher
Gestational age (weeks), median (IQR)	32.0 (31.7–32.0)	29.9 (28.7–31.9)	29.2 (27.2–29.9)	<0.001^*^	Kruskal–Wallis
Maternal parity, median (IQR)	1.0 (1.0–2.0)	1.0 (1.0–1.0)	1.0 (1.0–2.0)	0.7253	Kruskal–Wallis
Maternal gravidity, median (IQR)	3.0 (1.0–3.0)	1.0 (1.0–1.5)	2.0 (1.0–3.0)	0.1311	Kruskal–Wallis
Birth weight (kg), median (IQR)	1.760 (1.610–1.900)	1.510 (1.030–1.575)	1.075 (0.910–1.510)	<0.001^*^	Kruskal–Wallis
Apgar score (1 min), median (IQR)	8.0 (7.0–8.0)	7.0 (7.0–8.0)	6.0 (6.0–8.0)	0.0831	Kruskal–Wallis
Apgar score (5 min), median (IQR)	9.0 (8.0–9.0)	8.0 (8.0–9.0)	8.0 (8.0–9.0)	0.3137	Kruskal–Wallis

Values are expressed as median (IQR) or *n* (%). *p*-values were calculated using Kruskal–Wallis test for continuous variables and Chi-square or Fisher's exact test for categorical variables.

^*^P < 0.05 was considered statistically significant.

### 3.2 Alterations in bile acid profiles

**Figure 1:**
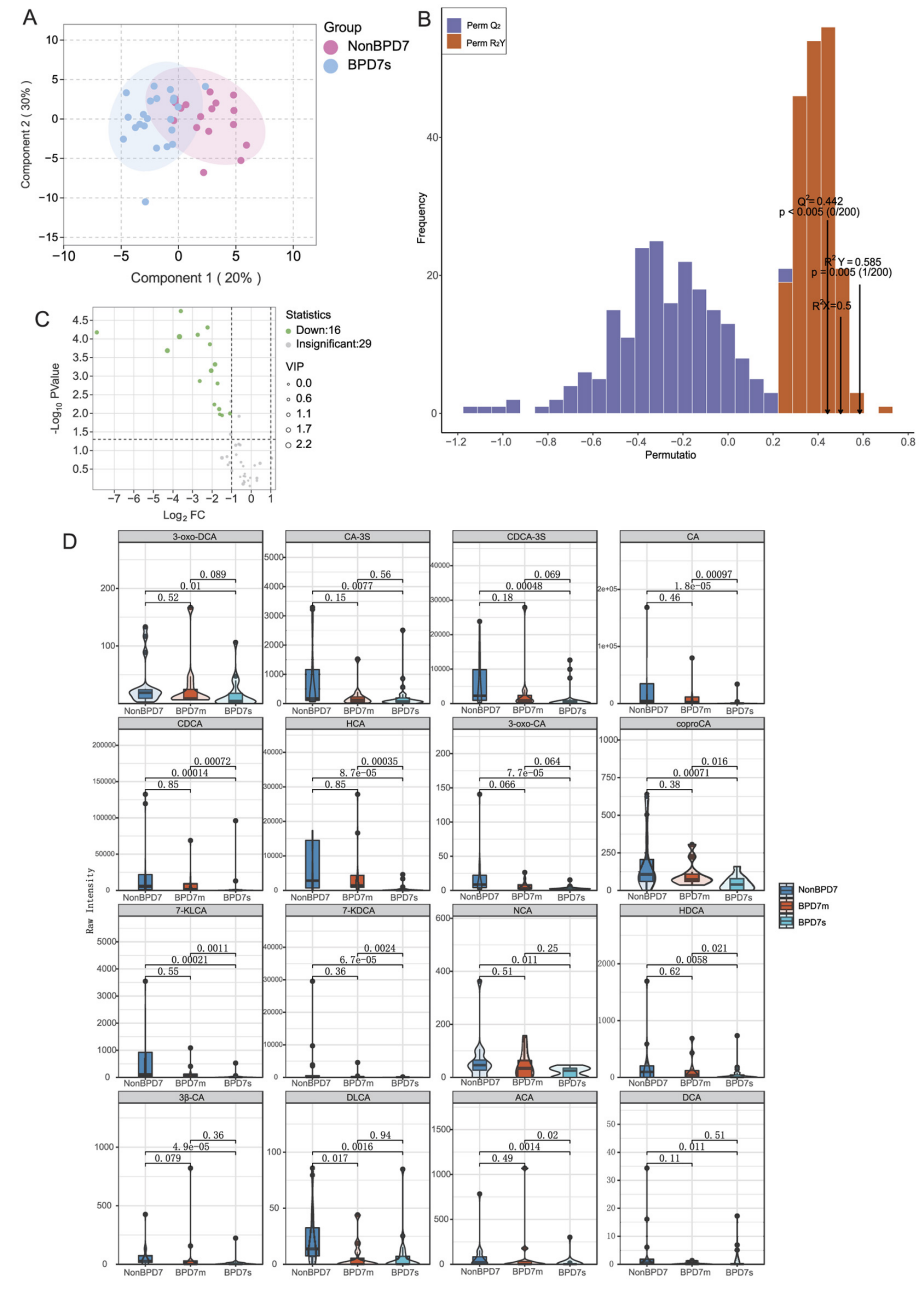
Targeted bile acid metabolomics in preterm infants with different BPD severities. **(A)** OPLS-DA score plot showing clear separation between NonBPD7 and Severe BPD (BPD7s) groups (Component 1: 20%; Component 2: 30%). **(B)** Permutation test validating model reliability (y-axis: frequency of permuted $R_{Y}^{2}$ and *Q*^2^ values). **(C)** Volcano plot identifying 16 significantly downregulated bile acids in BPD7s compared with NonBPD7 (VIP > 1, *P* < 0.05, |log_2_FC| > 1). **(D)** Violin plots with overlaid boxplots showing representative metabolites. BPD, bronchopulmonary dysplasia; NonBPD7, non-BPD group at postnatal day 7; BPD7m, non-severe BPD group (mild + moderate) at postnatal day 7; BPD7s, severe BPD group at postnatal day 7; 3-oxo-DCA, 3-oxodeoxycholic acid; CA-3S, cholic acid-3-sulfate; CDCA-3S, chenodeoxycholic acid-3-sulfate; CA, cholic acid; CDCA, chenodeoxycholic acid; HCA, hyocholic acid; 3-oxo-CA, 3-oxocholic acid; coproCA, coprocholic acid; 7-KLCA, 7-ketolithocholic acid; 7-KDCA, 7-ketodeoxycholic acid; NCA, norcholic acid; HDCA, hyodeoxycholic acid; 3β-CA, 3β-cholic acid; DLCA, deoxylithocholic acid; ACA, allocholic acid; DCA, deoxycholic acid; VIP, variable importance in projection; log_2_FC, log_2_ fold change. Primary bile acids: CA, CDCA, CA-3S, and CDCA-3S; Secondary bile acids: 3-oxo-DCA, HCA, 3-oxo-CA, coproCA, 7-KLCA, 7-KDCA, NCA, HDCA, 3β-CA, DLCA, ACA, and DCA.

To investigate early metabolic differences between preterm infants with and without BPD of varying severity, we performed multigroup multivariate statistical analyses. Orthogonal partial least squares discriminant analysis (OPLS–DA) revealed the clearest separation between the NonBPD7 and BPD7s groups (see Supplementary Figures S1–S3 for the three-group model and additional pairwise results). Component 1 and Component 2 explained 20 and 30% of the total variance, respectively (cumulative 50%). In the score plot, NonBPD7 samples clustered mainly within the negative range of Component 1, whereas BPD7s samples were distributed in the positive range, with additional separation along Component 2, reflecting distinct metabolic profiles. The model showed a satisfactory fit and predictive capacity ($R_{X}^{2}=0.50$, $R_{Y}^{2}=0.585$, *Q*^2^ = 0.442). Permutation tests (200 iterations) confirmed model reliability, as both *Q*^2^ and $R_{Y}^{2}$ values of the original model were significantly higher than those from permuted models (*P* < 0.005; Figures 1A, B).

Differential metabolite screening identified 16 bile acids significantly downregulated in BPD7s compared with NonBPD7, based on VIP >1, *P* < 0.05, and |log_2_FC|>1. These comprised four primary bile acids—cholic acid (CA), chenodeoxycholic acid (CDCA), and their sulfated conjugates CA-3S and CDCA-3S—and 12 secondary bile acids, including deoxycholic acid (DCA), deoxylithocholic acid (DLCA), hyodeoxycholic acid (HCA), 3-oxocholic acid (3-oxo-CA), 3β-cholic acid (3β-CA), 7-ketolithocholic acid (7-KLCA), norcholic acid (NCA), hyodeoxycholic acid (HDCA), allocholic acid (ACA), and coprocholic acid (coproCA), among others. Fold changes for these metabolites ranged from 0.004 to 0.472 (Figure 1C).

To visualize the distribution patterns of key differential metabolites among the groups, violin plots with overlaid boxplots were generated (Figure 1D). The results revealed significant inter-group differences in multiple bile acids, including primary bile acids (CA and CDCA) and secondary bile acids (7-KDCA, 7-KLCA, HCA, HDCA, ACA, and coproCA). Notably, 7-ketodeoxycholic acid (7-KDCA), 7-ketolithocholic acid (7-KLCA), and HCA were undetectable in most BPD7s samples, with median values of zero, which was consistent with the quantitative results in Supplementary Table S1. 7-KDCA showed the largest reduction (fold change = 0.004), accompanied by a high VIP score and a highly significant *P* value (*P* < 0.001). Overall, these differential metabolites demonstrated strong discriminatory potential across BPD severity levels.

Other pairwise comparisons, such as between NonBPD7 and BPD7m, also revealed metabolic differences, but both the number of altered metabolites and the magnitude of change were smaller than in the NonBPD7 vs. BPD7s comparison.

### 3.3 Gut microbiota diversity and differential taxa

**Figure 2:**
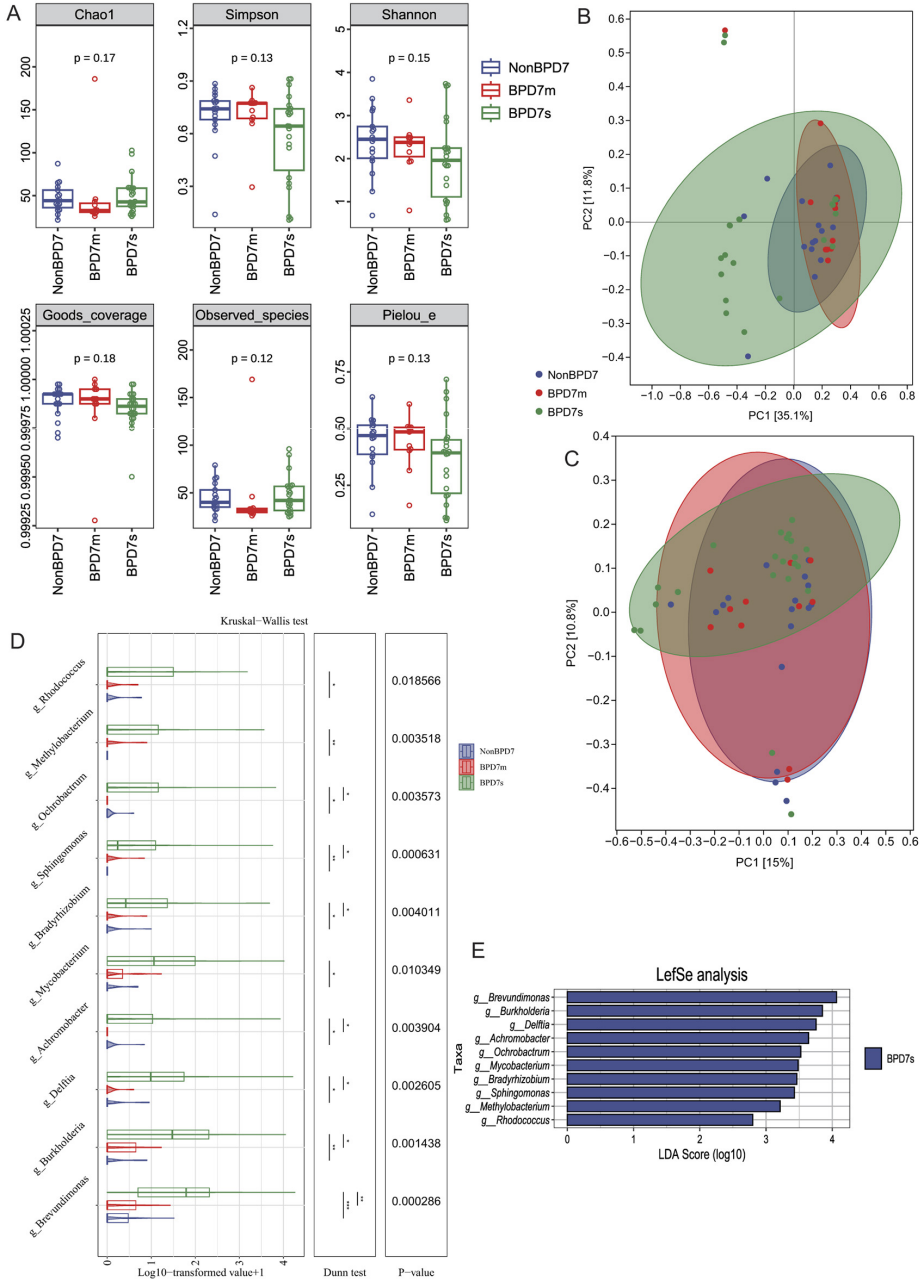
Gut microbiota composition on postnatal day 7 across BPD severities. **(A)** α-diversity indices (Chao1, Observed species, Shannon, Simpson, Pielou's evenness, Good's coverage) showed no significant differences among groups. **(B)** Principal coordinate analysis (PCoA) based on Bray–Curtis distances revealed clearer separation, while **(C)** unweighted UniFrac PCoA showed modest group differences. **(D)** Boxplots of log-transformed abundances of representative genera enriched in BPD7s. **(E)** LEfSe analysis identified ten genera with significant enrichment in BPD7s (LDA >2.0, *P* < 0.05), including *Brevundimonas, Burkholderia, Delftia, Achromobacter, Ochrobactrum, Mycobacterium, Bradyrhizobium, Sphingomonas, Methylobacterium*, and *Rhodococcus*. BPD, bronchopulmonary dysplasia; NonBPD7, non-BPD group at postnatal day 7; BPD7m, non-severe BPD group (mild + moderate) at postnatal day 7; BPD7s, severe BPD group at postnatal day 7; PCoA, principal coordinate analysis; LDA, linear discriminant analysis; LEfSe, linear discriminant analysis effect size. The symbols indicate statistical significance levels for the Dunn test as follows: *P < 0.05, **P < 0.01, and ***P < 0.001.

To characterize the gut microbiota on postnatal day 7 across BPD severities, we first assessed sequencing depth. Rarefaction curves plateaued in all groups and Good's coverage exceeded 0.99, indicating sufficient sampling (Supplementary Figure S4). Six α-diversity metrics—Chao1 (*P* = 0.17), Observed species (*P* = 0.12), Shannon (*P* = 0.15), Simpson (*P* = 0.13), Pielou's evenness (*P* = 0.13), and Good's coverage (*P* = 0.18)—did not differ among NonBPD7, BPD7m, and BPD7s (Figure 2A).

β-diversity (PERMANOVA) based on Bray–Curtis and unweighted UniFrac distances revealed overall between-group differences (Bray–Curtis: *R*^2^ = 0.1137, *P* = 0.001; unweighted UniFrac: *R*^2^ = 0.0551, *P* = 0.041; Supplementary Table S2; Supplementary Figures S5, S6), with clearer separation under Bray–Curtis. In the Bray–Curtis PCoA (Figure 2B), PC1 and PC2 explained 35.1 and 11.8% of the variance, respectively; NonBPD7 and BPD7m samples clustered mainly at positive PC1 values, whereas BPD7s samples were more dispersed. Ninety-five percent confidence ellipses supported the separation. For unweighted UniFrac (Figure 2C), PC1 and PC2 explained 15.0 and 10.8% of the variance.

LEfSe identified ten genera enriched in BPD7s (LDA>2.0, *P* < 0.05; Figure 2E): *Brevundimonas, Burkholderia, Delftia, Achromobacter, Ochrobactrum, Mycobacterium, Bradyrhizobium, Sphingomonas, Methylobacterium*, and *Rhodococcus*. Log-transformed abundances of these genera are shown as boxplots (Figure 2D). To confirm the robustness of these findings, Kruskal–Wallis tests were performed across the same taxa, indicating significant differences among groups (*P* < 0.05). Dunn's *post-hoc* tests showed the largest contrasts between NonBPD7 and BPD7s (all *P* < 0.05), followed by BPD7m vs. BPD7s (7/10 genera, *P* < 0.05), with no differences between NonBPD7 and BPD7m. Abundances generally followed BPD7s > BPD7m > NonBPD7.

Functional prediction analysis using PICRUSt2 was further performed to infer microbial metabolic potential. A total of 170 KEGG pathways were identified across all samples (Supplementary Table S3). While several metabolic pathways showed groupwise differences, pathways associated with bile acid metabolism, including primary and secondary bile acid biosynthesis (ko00120 and ko00121), did not differ significantly among groups.

### 3.4 Interactions between bile acids and gut microbiota

**Figure 3:**
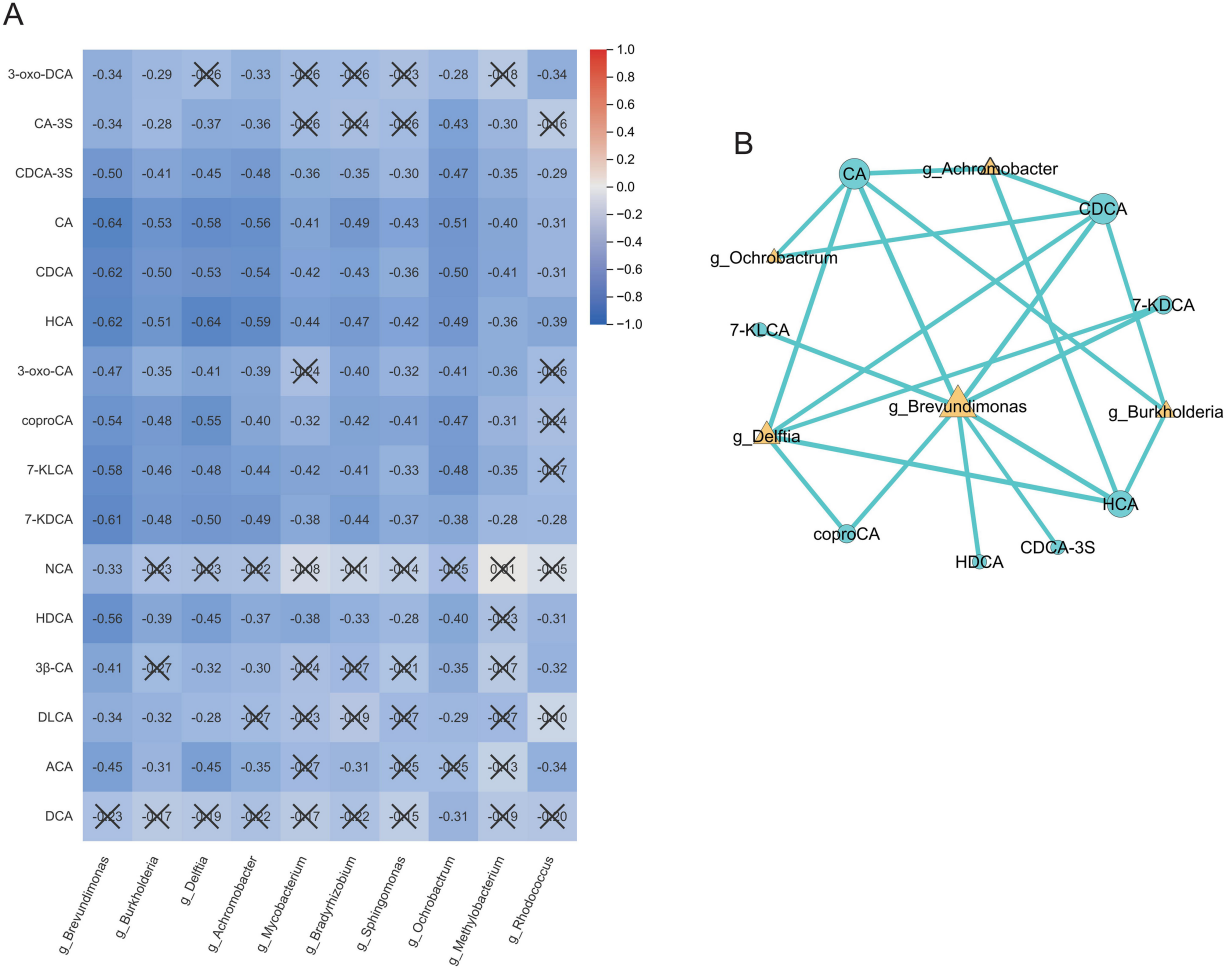
Correlation analysis of bile acids and microbial genera. **(A)** Spearman correlation heatmap showing predominantly negative associations between differential bile acids and bacterial genera. **(B)** Correlation network highlighting *Brevundimonas* as a central node linked to multiple bile acids. Significant correlations were defined as |ρ|>0.5 and *P* < 0.05. Cells marked with “ × ” indicate non-significant correlations (*P*≥0.05). The color scale represents the Spearman correlation coefficient (ρ), ranging from −1 (blue, strong negative) to +1 (red, strong positive). BPD, bronchopulmonary dysplasia; NonBPD7, non-BPD group at postnatal day 7; BPD7m, non-severe BPD group (mild + moderate) at postnatal day 7; BPD7s, severe BPD group at postnatal day 7; 3-oxo-DCA, 3-oxodeoxycholic acid; CA-3S, cholic acid-3-sulfate; CDCA-3S, chenodeoxycholic acid-3-sulfate; CA, cholic acid; CDCA, chenodeoxycholic acid; HCA, hyocholic acid; 3-oxo-CA, 3-oxocholic acid; coproCA, coprocholic acid; 7-KLCA, 7-ketolithocholic acid; 7-KDCA, 7-ketodeoxycholic acid; NCA, norcholic acid; HDCA, hyodeoxycholic acid; 3β-CA, 3β-cholic acid; DLCA, deoxylithocholic acid; ACA, allocholic acid; DCA, deoxycholic acid; ρ, Spearman correlation coefficient.

Spearman correlation analysis between the differentially abundant bile acids and microbial genera identified previously revealed a general pattern of negative associations, as shown in the heatmap (Figure 3A). Notably, the genus *Brevundimonas* was negatively correlated with 15 out of the 16 bile acids examined, while several bile acids—including CDCA-3S, HCA, CA, and CDCA—also exhibited consistent negative correlations with all the differential bacterial genera. Applying correlation thresholds of |ρ|>0.5 and *P* < 0.05, we found that multiple primary and secondary bile acids—namely CA, CDCA, CDCA-3S, coproCA, 7-KLCA, 7-KDCA, HDCA, and HCA—were significantly negatively correlated with proteobacterial genera such as *Brevundimonas* and *Delftia* (Supplementary Table S4). Among these, the strongest negative correlations were observed between CA and *Brevundimonas* (ρ = −0.64), HCA and *Delftia* (ρ = −0.64), and CDCA and *Brevundimonas* (ρ = −0.62). Correlation network analysis (Figure 3B) further indicated that *Brevundimonas* occupies a central position within the network, exhibiting direct interactions with all eight bile acids included in the analysis.

### 3.5 Machine learning-based prediction modeling

**Figure 4:**
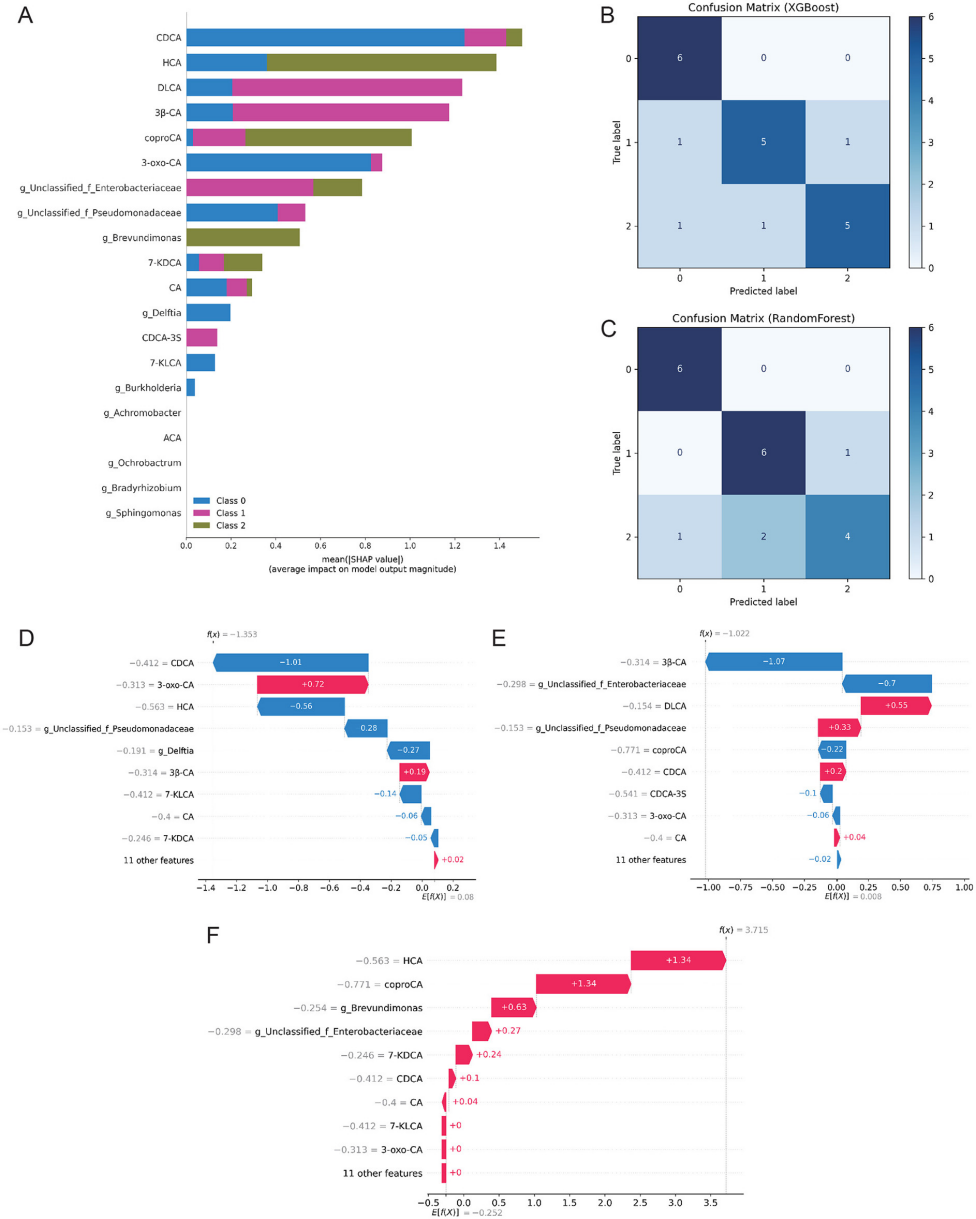
Machine learning models for BPD severity classification. **(A)** Global feature importance and SHAP summary plot for the selected feature set. **(B)** Confusion matrix of the XGBoost classifier on the independent test set (rows: true labels; columns: predicted labels). **(C)** Confusion matrix of the Random Forest classifier on the independent test set (same label order as in panel B). **(D–F)** Individual SHAP waterfall plots illustrating feature contributions to representative cases from each class (D = NonBPD7, E = BPD7m, F = BPD7s). Models were trained with SMOTE; evaluation used five-fold stratified cross-validation and an independent test set. Class labels were encoded as 0 = NonBPD7, 1 = BPD7m, and 2 = BPD7s. BPD, bronchopulmonary dysplasia; NonBPD7, non-BPD group at postnatal day 7; BPD7m, non-severe BPD group (mild + moderate) at postnatal day 7; BPD7s, severe BPD group at postnatal day 7; SHAP, SHapley Additive exPlanations; CDCA, chenodeoxycholic acid; HCA, hyocholic acid; DLCA, deoxylithocholic acid; 7-KDCA, 7-ketodeoxycholic acid; 3β-CA, 3β-cholic acid; coproCA, coprocholic acid; 3-oxo-CA, 3-oxocholic acid; CA, cholic acid; CDCA-3S, chenodeoxycholic acid-3-sulfate; 7-KLCA, 7-ketolithocholic acid; ACA, allocholic acid.

Using Kruskal–Wallis testing with FDR adjustment, 20 differential features (bile acids and Proteobacteria-related genera) were selected. Class imbalance in the training set was addressed with SMOTE. Global feature importance and SHAP analysis (Figure 4A) highlighted bile acids such as CDCA, HCA, and DLCA, together with bacterial taxa including unclassified Enterobacteriaceae, *Brevundimonas*, and *Delftia*, as the major contributors to the three-class discrimination. Feature importance and SHAP analyses indicated strong contributions of these metabolites and taxa to three-class discrimination.

Tree-based models performed best in five-fold cross-validation: RF achieved a macro-F1 of 0.78 and XGBoost 0.76. Corresponding AUCs were 0.93 and 0.91, outperforming LR, NB, KNN, and SVM (AUC <0.90) (Supplementary Table S5). On the independent test set (70/30 split), both models reached 80% accuracy. Recall for the Non-BPD class approached 100%, whereas misclassification occurred primarily between Mild and Severe BPD (Figures 4B, C). Model fusion did not yield further gains (Supplementary Figures S7, S8). Among the two tree-based classifiers, XGBoost was ultimately chosen for subsequent SHAP analysis, owing to its consistently robust cross-validation performance, reliable probability calibration, and well-established interpretability in biomedical applications.

Robustness checks—including repeated stratified splits and permutation testing—supported performance exceeding chance (*p*≈0.001). Stability analysis further confirmed that the set of top-20 SHAP-ranked features was highly reproducible (mean Jaccard ≈1.00) (Supplementary Figures S9–S11). Individual-level SHAP waterfall plots (Figures 4D–F) revealed that alterations in bile acids and specific bacterial taxa were critical determinants for class prediction, consistent with the global SHAP ranking. Notably, CDCA, HCA, and *Brevundimonas* consistently emerged as the most stable contributors across all resampling runs (Supplementary Figure S8).

## 4 Discussion

This study integrated analyses of clinical features, fecal bile acid profiles, and gut microbiome structure in 50 preterm infants. We confirmed that the severity of bronchopulmonary dysplasia (BPD) was inversely correlated with gestational age and birth weight, consistent with previous reports (Klinger et al., 2013; Geetha et al., 2021). These findings not only reaffirm that developmental immaturity is a prerequisite for BPD, but also suggest that such immaturity may increase susceptibility to microbiome–metabolite dysregulation by compromising the host's ability to maintain microenvironmental homeostasis. We focused on the 7th day after birth as a critical time point—a period within the initial colonization window (typically the first 1–2 weeks of life), during which the gut microbiome exhibits high volatility (Ning et al., 2025; Zhang, 2022). At this early stage, infants who later developed severe BPD already showed significant differences in metabolic and microbial profiles compared to non-BPD infants. This temporal alignment suggests that early microenvironmental disruption is not merely a consequence of disease progression, but may actively contribute to pathological processes during a critical period of lung development. This relationship is particularly salient in preterm infants, in whom immature gut barrier function—marked by reduced expression of tight junction proteins such as occludin (Golubkova and Hunter, 2023)—coincides with delayed hepatic maturation of UDP-glucuronosyltransferase activity (Kawade and Onishi, 1981), impairing metabolic clearance. Against a backdrop of immature immune function (Collado et al., 2015), these factors collectively diminish the host's capacity to maintain homeostasis in response to microbial and metabolic challenges.

The intestinal microbiota plays a central role in maintaining bile-acid compositional homeostasis by reshaping the host bile-acid pool through deconjugation, dehydroxylation, and dehydrogenation reactions (Ikegami and Honda, 2018). Bile salt hydrolases are widely distributed across bacterial phyla, while C-7 hydroxysteroid dehydrogenases (7α/7β-HSDH) and the downstream 7α-dehydroxylation pathway are predominantly encoded by obligate anaerobes within the class *Clostridia* (e.g., *Clostridium, Eubacterium*); members of Bacteroidetes (e.g., *Bacteroides* spp.) have also been reported to harbor BSH and certain HSDH activities (Doden and Ridlon, 2021). These enzymes oxidize primary bile acids to 7-keto intermediates (e.g., 7-oxo-DCA, 7-oxo-LCA), which are subsequently converted by *bai*-operon–positive strains via 7α-dehydroxylation into secondary bile acids such as DCA and LCA (Wise and Cummings, 2022). In the metabolic profiles of infants with severe BPD, we observed an overall reduction in bile acid levels. Notably, 7-keto bile acids such as 7-KDCA and 7-KLCA were largely undetectable or present at low abundance within the current detection limits. This pattern is consistent with a potential deficiency in specific microbial metabolic functions. However, it may also reflect a general reduction in the bile acid pool size and/or the immaturity of relevant microbial niches in early life. Therefore, these observations cannot be solely attributed to overall changes in microbial abundance (Doden and Ridlon, 2021; Guzior and Quinn, 2021; Bennett et al., 2003).

Concurrently, infants with severe BPD exhibited significantly reduced levels of sulfated bile acids. Sulfonation enhances bile acid solubility, facilitating their excretion via urine and feces, reducing enterohepatic recirculation, and thereby helping to regulate the total bile acid pool and mitigate the accumulation of toxic hydrophobic bile acids (Alnouti, 2009). This reduction may suggest not only potentially impaired host sulfotransferase activity or enhanced microbial desulfation (Assem et al., 2004; Ridlon et al., 2016), but could also reflect broader influences such as a reduced overall bile acid pool or altered excretion kinetics.

Our findings reveal a broad reduction in fecal bile acid levels in infants who later developed severe BPD. This observation aligns with the established role of bile acids as signaling molecules that may influence immune and inflammatory pathways via receptors such as FXR and TGR5 (De Luca, 2022; Li et al., 2015). While preclinical models suggest that physiological bile acid signaling helps regulate pulmonary inflammation and vascular integrity (De Luca, 2022; Cao, 2024; Wang et al., 2011), our study cannot establish a direct mechanistic link in humans. Instead, our data provide novel clinical evidence of an association between early-life bile acid deficiency and subsequent BPD development. This deficiency could hypothetically impair FXR- and TGR5-mediated anti-inflammatory and pro-developmental signals, thereby increasing susceptibility to lung injury, but this requires functional validation. It is also important to note that while abnormal pulmonary accumulation of bile acids has been shown to exert direct cytotoxic effects (Zecca et al., 2008; De Luca, 2022; Chen et al., 2016), our metabolomic analysis of fecal samples suggests that systemic deficiency, rather than excess, is the predominant early-life alteration associated with BPD risk. We therefore propose that an early reduction in bile acids may represent a novel, clinically relevant risk marker for BPD, and that the potential disruption of bile acid signaling pathways represents a plausible, though not yet proven, contributing mechanism to its pathogenesis.

Consistent with the metabolic findings, infants who later developed severe BPD already exhibited significant alterations in their gut microbiome structure (beta diversity) by day 7 of life, although alpha diversity remained comparable to that of the non-BPD group. Notably, we observed an increased abundance of several opportunistic pathogens—including *Brevundimonas, Delftia*, and *Achromobacter*—genera previously associated with neonatal sepsis and other serious infections (Viswanathan et al., 2010; Scaglione, 2025; Ozden Turel, 2013). Emerging evidence suggests that colonization by these taxa may impair intestinal barrier and immune function: aberrant microbial colonization in preterm infants has been linked to disrupted immune maturation (Yao et al., 2023); experimental work indicates that intestinal colonization with *Brevundimonas vesicularis* can aggravate inflammatory responses and reduce efficacy of immunomodulatory treatments (Liu, 2023); and studies of milk microbiota have reported correlations between overgrowth of *Brevundimonas* and metabolic or inflammatory dysregulation (Liu, 2024). These findings align with our results and suggest that such organisms may contribute to early disruptions in intestinal homeostasis, potentially influencing BPD pathogenesis.

Integrated analysis revealed broad negative correlations between several downregulated bile acids and the enriched bacterial taxa. This aligns with the established view that reduced bile acid levels diminish antimicrobial pressure, thereby providing a competitive advantage to opportunistic pathogens (Larabi et al., 2023; Lynch, 2023). Notably, *Brevundimonas* occupied a central role in the bile acid–microbiota interaction network, showing negative correlations with all differential bile acids. This suggests that it may not merely be a passive consequence of bile acid deficiency, but also contribute to driving dysbiosis and amplifying inflammatory responses.

Based on these findings, we developed and validated an early prediction model integrating both bile acid and microbial features to assess its ability to stratify risk and identify severe BPD as early as day 7 after birth. Using five-fold cross-validation and an independent test set (70/30 split), both XGBoost and RF models demonstrated consistent performance, with overall accuracy around 80% and a maximum AUC of 0.91 for severe BPD. The models exhibited high specificity in identifying non-BPD infants, approaching 100%, indicating high reliability in ruling out non-BPD cases. These results are consistent with recent similar studies (Choi, 2025).

Compared to previous models primarily reliant on conventional clinical indicators such as gestational age and birth weight (Choi, 2025; Tao, 2024), we developed a multi-omics-based interpretable model that not only demonstrates superior predictive performance but also provides mechanistic insights into disease phenotypes by integrating bile acid and microbial features to stratify risk and identify severe BPD as early as day 7 after birth. SHAP-based interpretability analysis revealed that the top-ranking features contributing to model predictions overlapped with previously identified differential metabolites and microbial genera. In particular, CDCA, HCA, and *Brevundimonas* emerged as the most stable contributors across repeated resampling, while additional factors such as DLCA and *Delftia* also showed relevance. Although the sets of features highlighted by SHAP (which reflects global feature importance on model output) and correlation analysis (which captures linear associations between variables) are not identical, both methods underscore the importance of several key biomarkers. This finding aligns with the common use of SHAP for identifying critical biomarkers in multi-omics studies (Liu et al., 2025), and further supports the potential role of these factors in BPD pathogenesis.

Furthermore, robustness evaluations—including permutation tests and feature stability analysis—demonstrated that the model significantly outperformed random chance and that the most influential features were reproducible across subsampling. This study confirms that integrating fecal bile acid metabolome and gut microbiome data within an interpretable tree-based modeling framework (XGBoost–SHAP) yields an early discrimination strategy with both high predictive performance and biological interpretability. This approach aligns with advanced multi-omics strategies for studying neonatal diseases (Chen, 2024; Xu, 2022; Ding, 2025) and highlights the potential of this methodology for very early risk prediction and mechanistic investigation in BPD.

In summary, this study revealed an early co-occurrence of bile acid homeostasis disruption and microbial dysbiosis within the 7-day postnatal window, and demonstrated the feasibility of an interpretable machine-learning model for early BPD risk prediction.

This study has several limitations. First, the modest sample size (*n* = 50) and single-center design constrain statistical power and may introduce selection bias, limiting external validity and generalizability. Second, the observational nature of the study precludes causal inference between bile acid–microbiota interactions and BPD severity, and residual confounding cannot be fully excluded. Third, although the machine-learning models performed well under internal validation, their transportability requires confirmation in larger, multicenter prospective cohorts, including rigorous assessments of calibration and overfitting. Finally, 16S rRNA gene sequencing offers limited species-level resolution and provides only indirect functional inference, which may restrict interpretation of microbial functional potential relative to shotgun metagenomics or metatranscriptomics.

## 5 Conclusion

**Figure 5:**
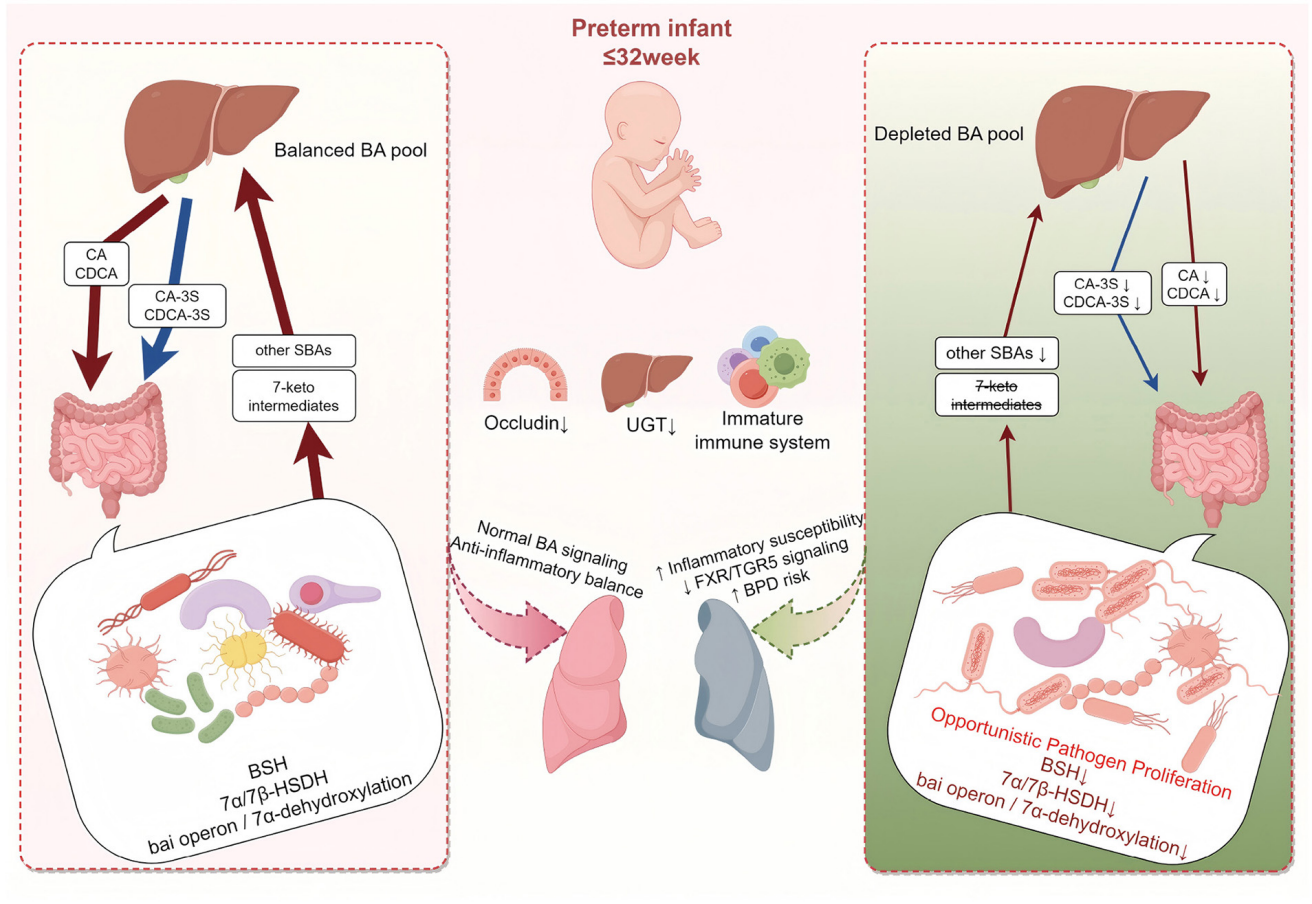
Schematic of the gut bile acid-microbiota axis and BPD risk in preterm infants. At day 7 of life, all infants born ≤ 32 weeks share prematurity-related immaturity–reduced intestinal barrier (Occludin↓), delayed hepatic UGT activity, and an immature immune system–which constitutes a common background vulnerability. **(Left)** despite this baseline, some infants remain relatively balanced; microbial BSH, 7α/7β-HSDH, and the *bai* operon sustain conversion of CA/CDCA into 7-keto intermediates and secondary bile acids, preserving a balanced bile acid pool and FXR/TGR5-mediated anti-inflammatory signaling. **(Right)** under the same baseline, a subset shows early dysregulation with overgrowth of opportunistic taxa (e.g., *Brevundimonas, Delftia, Achromobacter*), loss of key anaerobic functions, and marked reductions across the bile-acid spectrum–including primary, secondary, sulfated, and 7-oxo/7-keto species–resulting in a depleted bile-acid pool and weakened FXR/TGR5 signaling, thereby increasing inflammatory susceptibility and BPD risk during lung development. This is a conceptual model and does not imply proven causality. Arrow thickness represents the relative size of the bile-acid pool (thinner = reduced pool). BA, bile acid; SBA, secondary bile acid; CA, cholic acid; CDCA, chenodeoxycholic acid; CA-3S, cholic acid-3-sulfate; CDCA-3S, chenodeoxycholic acid-3-sulfate; UGT, UDP-glucuronosyltransferase; BSH, bile salt hydrolases; HSDH, hydroxysteroid dehydrogenases; FXR, Farnesoid X Receptor; TGR5, Takeda G-protein-coupled Receptor 5; BPD, bronchopulmonary dysplasia.

In a clinical cohort of preterm infants, we show that severe BPD is preceded by early disruption of the gut bile acid–microbiota axis, characterized by a marked reduction in bile acid levels and enrichment of opportunistic proteobacterial taxa. These findings provide clinical support for the gut–lung axis framework, implicating bile acid–microbiota crosstalk in the early pathogenesis of BPD. As illustrated in Figure 5, this conceptual model summarizes the proposed gut bile acid-microbiota-lung axis and its potential role in BPD pathogenesis. Building on this biology, an interpretable multi-omics model (XGBoost with SHAP) accurately predicted BPD severity and prioritized bile acids (e.g., CDCA, HCA) and the genus *Brevundimonas* as the most stable contributors. The work identifies candidate biomarker panels for early risk stratification and offers mechanistic insight beyond traditional inflammation-centric views. Future studies should validate these results in large, prospective, multicenter cohorts and evaluate targeted interventions that modulate the bile acid–microbiota axis for prevention or attenuation of BPD.

## Data Availability

The datasets presented in this study can be found in online repositories. The names of the repository/repositories and accession number(s) can be found at: https://www.ncbi.nlm.nih.gov/, PRJNA1328652.
