# Supplementary material for: Disruption of the gut bile acid-microbiota axis precedes severe bronchopulmonary dysplasia in preterm infants
Source: Front Microbiol. 2025 Nov 24;16:1705965. doi: 10.3389/fmicb.2025.1705965 (PMC12682640; doi:10.3389/fmicb.2025.1705965)
Supplement: Supplementary file 1 [file Data_Sheet_1.pdf]

frontiers

Supplementary Material

1 SUPPLEMENTARY TABLES AND FIGURES

1.1 Tables

Table S1. Differential bile acid metabolites across groups.(Main Table)

Index	Mean ± SD (BPD7s)	Mean ± SD (BPD7m)	Mean ± SD (NonBPD7)	Median (BPD7s)	Median (BPD7m)	Median (NonBPD7)
3-oxo-CA	3.022 ± 3.207	7.337 ± 7.871	20.083 ± 33.118	2.285	3.301	8.734
3-oxo-DCA	15.252 ± 24.553	29.239 ± 47.486	32.289 ± 39.926	4.268	8.851	18.810
3β-CA	14.818 ± 47.094	95.392 ± 245.146	69.577 ± 102.564	0.000	0.000	28.316
7-KDCA	11.967 ± 33.450	480.542 ± 1360.541	2840.075 ± 7328.004	0.000	47.659	74.297
7-KLCA	34.496 ± 111.517	175.111 ± 324.103	672.607 ± 1014.696	0.000	35.326	105.553
ACA	14.312 ± 64.064	120.284 ± 319.504	88.608 ± 187.529	0.000	0.000	21.998
CA	2006.451 ± 7187.830	11679.483 ± 23252.578	24295.204 ± 43258.699	130.919	2435.176	4317.202
CA-3S	260.725 ± 541.938	265.182 ± 433.170	810.983 ± 1130.519	71.806	125.492	175.768
CDCA	5163.579 ± 20496.389	10324.885 ± 20212.848	22297.122 ± 40389.561	64.547	1562.582	5922.222
CDCA-3S	1726.593 ± 3468.026	3738.908 ± 8110.713	6227.904 ± 7389.859	410.006	902.719	2275.662
coproCA	1.329 ± 3.979	0.316 ± 0.547	3.726 ± 8.836	0.000	0.000	0.543
DCA	7.265 ± 18.552	6.710 ± 13.869	23.953 ± 26.362	0.000	0.000	13.868
DLCA	507.148 ± 1169.933	5446.032 ± 8798.496	6443.734 ± 7039.577	69.712	1378.325	2852.632
HCA	59.688 ± 158.824	137.941 ± 220.001	220.159 ± 407.938	0.000	41.345	95.665
HDCA	20.506 ± 18.388	45.312 ± 52.593	62.544 ± 84.423	25.536	33.546	46.223
NCA	46.449 ± 48.183	106.260 ± 84.490	191.844 ± 205.722	39.916	74.909	108.063

Values are expressed as mean ± SD and median. Variable importance in projection (VIP) scores, p-values, fold change, and $\log_2$ fold change ($\log_2$ FC) are provided for pairwise comparisons (NonBPD7 vs. BPD7m, BPD7m vs. BPD7s, and NonBPD7 vs. BPD7s). Differential metabolites were defined as VIP > 1, $p < 0.05$, and $|\log_2 \text{FC}| > 1$.

Table S1. Differential bile acid metabolites across groups.(Sub-table 1)

Index	NonBPD7 vs BPD7m VIP	NonBPD7 vs BPD7m P-value	NonBPD7 vs BPD7m Fold Change	NonBPD7 vs BPD7m log_2FC	BPD7m vs BPD7s VIP	BPD7m vs BPD7s P-value
3-oxo-CA	1.4414	0.0659	0.3653	-1.4527	1.7324	0.0638
3-oxo-DCA	0.7678	0.5165	0.9055	-0.1432	1.1338	0.0894
3β-CA	0.7258	0.0794	1.3710	0.4553	1.5751	0.3615
7-KDCA	1.2007	0.3567	0.1692	-2.5632	1.2990	0.0024
7-KLCA	1.7789	0.5471	0.2603	-1.9415	1.4829	0.0011
ACA	0.5932	0.4877	1.3575	0.4409	1.5820	0.0202
CA	0.9392	0.4581	0.4807	-1.0567	1.6243	0.0010
CA-3S	1.4264	0.1469	0.3270	-1.6127	0.2143	0.5596
CDCA	1.0577	0.8533	0.4631	-1.1107	0.7708	0.0007
CDCA-3S	0.6570	0.1755	0.6003	-0.7361	0.9627	0.0692
coproCA	1.7558	0.3777	0.5539	-0.8523	1.2536	0.0165
DCA	1.5423	0.1130	0.0847	-3.5615	0.6404	0.5139
DLCA	2.2881	0.0174	0.2801	-1.8359	0.2673	0.9439
HCA	0.2036	0.8533	0.8452	-0.2427	2.0046	0.0004
HDCA	0.5199	0.6209	0.6265	-0.6745	1.0299	0.0212
NCA	0.6884	0.5052	0.7245	-0.4650	1.2079	0.2450

Values are expressed as mean $\pm$ SD and median. Variable importance in projection (VIP) scores, p-values, fold change, and $\log_2$ fold change ($\log_2$ FC) are provided for pairwise comparisons (NonBPD7 vs. BPD7m, BPD7m vs. BPD7s, and NonBPD7 vs. BPD7s). Differential metabolites were defined as VIP > 1, $p < 0.05$, and $|\log_2 \text{FC}| > 1$.

Table S1. Differential bile acid metabolites across groups.(Sub-table 2)

Index	BPD7m vs BPD7s Fold Change	BPD7m vs BPD7s log_2FC	NonBPD7 vs BPD7s VIP	NonBPD7 vs BPD7s P-value	NonBPD7 vs BPD7s Fold Change	NonBPD7 vs BPD7s log_2FC
3-oxo-CA	0.4118	-1.2799	1.3721	0.0001	0.1505	-2.7326
3-oxo-DCA	0.5216	-0.9389	1.4943	0.0101	0.4724	-1.0820
3β-CA	0.1553	-2.6865	1.6902	0.0000	0.2130	-2.2312
7-KDCA	0.0249	-5.3275	1.4911	0.0001	0.0042	-7.8907
7-KLCA	0.1970	-2.3438	1.3795	0.0002	0.0513	-4.2853
ACA	0.1190	-3.0711	2.2059	0.0014	0.1615	-2.6302
CA	0.1718	-2.5413	1.5271	0.0000	0.0826	-3.5980
CA-3S	0.9832	-0.0245	1.7941	0.0077	0.3215	-1.6371
CDCA	0.5001	-0.9997	1.9926	0.0001	0.2316	-2.1104
CDCA-3S	0.4618	-1.1147	1.4610	0.0005	0.2772	-1.8508
coproCA	0.4371	-1.1939	1.0152	0.0007	0.2421	-2.0462
DCA	4.2124	2.0747	1.1993	0.0114	0.3568	-1.4869
DLCA	1.0828	0.1147	1.4890	0.0016	0.3033	-1.7212
HCA	0.0931	-3.4247	1.2749	0.0001	0.0787	-3.6674
HDCA	0.4327	-1.2085	1.2541	0.0058	0.2711	-1.8830
NCA	0.4526	-1.1438	1.0428	0.0106	0.3279	-1.6088

Values are expressed as mean $\pm$ SD and median. Variable importance in projection (VIP) scores, p-values, fold change, and $\log_2$ fold change ($\log_2$ FC) are provided for pairwise comparisons (NonBPD7 vs. BPD7m, BPD7m vs. BPD7s, and NonBPD7 vs. BPD7s). Differential metabolites were defined as VIP > 1, $p < 0.05$, and $|\log_2 \text{FC}| > 1$.

Table S2. β-diversity analysis results based on Bray–Curtis and unweighted UniFrac distances.

Distance metric	Test type	Group	F	R^2	P-value	Padj (BH)
Bray–Curtis	PERMANOVA	All groups	3.0137	0.1137	0.001^{***}	—
	Dispersion (Kruskal–Wallis)	All groups	—	—	$8.02 \times 10^{-58****}$	8.02×10^{-58}
	Dispersion (Kruskal–Wallis)	NonBPD7	—	—	$1.15 \times 10^{-30****}$	1.15×10^{-30}
	Dispersion (Kruskal–Wallis)	BPD7m	—	—	$3.26 \times 10^{-15****}$	3.26×10^{-15}
	Dispersion (Kruskal–Wallis)	BPD7s	—	—	0.0217^{*}	0.0217
Unweighted UniFrac	PERMANOVA	All groups	1.3704	0.0551	0.041^{*}	—
	Dispersion (Kruskal–Wallis)	All groups	—	—	0.1073	0.1073
	Dispersion (Kruskal–Wallis)	NonBPD7	—	—	0.0673	0.0673
	Dispersion (Kruskal–Wallis)	BPD7m	—	—	0.2479	0.2479
	Dispersion (Kruskal–Wallis)	BPD7s	—	—	0.7174	0.7174

The table summarizes PERMANOVA (adonis function) for group differences (F, R^2, p-value) and homogeneity-of-dispersion tests using `betadisper` with Kruskal–Wallis (Padjust, BH correction). Significance levels: ns $P > 0.05$, * $P \leq 0.05$, ** $P \leq 0.01$, *** $P \leq 0.001$, **** $P \leq 0.0001$.

Table S3: Predicted KEGG metabolic pathways inferred by PICRUSt2 across all samples ($n = 170$).

var	description	p.value	adj.p.value
ko00010	Glycolysis / Gluconeogenesis	0.0230943774857638	0.10632246871878
ko00020	Citrate cycle (TCA cycle)	0.009533880763282	0.0686048857063766
ko00030	Pentose phosphate pathway	0.0417363632316043	0.127359054140066
ko00040	Pentose and glucuronate interconversions	0.037506538073256	0.127240437844916
ko00051	Fructose and mannose metabolism	0.00504450471360308	0.0464880871102599
ko00052	Galactose metabolism	0.0748929512476876	0.171526154366017
ko00053	Ascorbate and aldarate metabolism	0.0409836036899306	0.127359054140066
ko00061	Fatty acid biosynthesis	0.297549809979275	0.417624324866361
ko00071	Fatty acid metabolism	0.0054691867188541	0.0464880871102599
ko00072	Synthesis and degradation of ketone bodies	0.00706700620605195	0.0572090978585158
ko00100	Steroid biosynthesis	0.0189967577775255	0.0978620855205861
ko00120	Primary bile acid biosynthesis	0.241554396948602	0.366645066796985
ko00121	Secondary bile acid biosynthesis	0.141910683602804	0.259406625940609
ko00130	Ubiquinone and other terpenoid-quinone biosynthesis	0.479693285716307	0.575367622591575
ko00140	Steroid hormone biosynthesis	0.298312242015539	0.417624324866361
ko00190	Oxidative phosphorylation	0.0246527006002482	0.109574103695252

Continued on next page

Table S3 continued from previous page

var	description	p.value	adj.p.value
ko00195	Photosynthesis	0.399486102669451	0.499357628336813
ko00196	Photosynthesis - antenna proteins	0.603247844083788	0.657385471116948
ko00230	Purine metabolism	0.659569053492264	0.700792119335531
ko00240	Pyrimidine metabolism	0.496979104070856	0.578674299260585
ko00250	Alanine, aspartate and glutamate metabolism	0.0404426483135852	0.127359054140066
ko00253	Tetracycline biosynthesis	0.0403961979802587	0.127359054140066
ko00260	Glycine, serine and threonine metabolism	0.00248109246178922	0.0464880871102599
ko00270	Cysteine and methionine metabolism	0.0988471501403262	0.209508345444897
ko00280	Valine, leucine and isoleucine degradation	0.0169788837731817	0.0931100077884157
ko00281	Geraniol degradation	0.0742738958292094	0.171526154366017
ko00290	Valine, leucine and isoleucine biosynthesis	0.0291371642105129	0.111792684738699
ko00300	Lysine biosynthesis	0.200051791771061	0.323893377153147
ko00310	Lysine degradation	0.0251375884947931	0.109574103695252
ko00311	Penicillin and cephalosporin biosynthesis	0.0490900568321667	0.130395463460443
ko00330	Arginine and proline metabolism	0.0457443492526082	0.129608989549056
ko00340	Histidine metabolism	0.00771632948615926	0.0596261823930489
ko00350	Tyrosine metabolism	0.0981703891879652	0.209508345444897
ko00360	Phenylalanine metabolism	0.144269206205987	0.260912394202317
ko00361	Chlorocyclohexane and chlorobenzene degradation	0.72484364608463	0.750384677730679
ko00362	Benzoate degradation	0.628897272415483	0.675995999775574
ko00363	Bisphenol degradation	0.0279047762998019	0.111792684738699
ko00364	Fluorobenzoate degradation	0.0301635524326681	0.111792684738699
ko00380	Tryptophan metabolism	0.0345143753616957	0.119743751254862
ko00400	Phenylalanine, tyrosine and tryptophan biosynthesis	0.00411644636556167	0.0464880871102599
ko00410	beta-Alanine metabolism	0.00537186223300505	0.0464880871102599
ko00430	Taurine and hypotaurine metabolism	0.110074313985059	0.222551364417304
ko00440	Phosphonate and phosphinate metabolism	0.305649537049829	0.417624324866361
ko00450	Selenocompound metabolism	0.717669708333361	0.74848987985688
ko00460	Cyanoamino acid metabolism	0.0130647232023844	0.0822593683113095
ko00471	D-Glutamine and D-glutamate metabolism	0.303791887125052	0.417624324866361
ko00472	D-Arginine and D-ornithine metabolism	0.0177211501423352	0.0941436101311558
ko00473	D-Alanine metabolism	0.370571674739701	0.477251399285978
ko00480	Glutathione metabolism	0.176760415101784	0.298761104328536
ko00500	Starch and sucrose metabolism	0.326791322382962	0.434019725039872
ko00510	N-Glycan biosynthesis	0.00220344080783684	0.0464880871102599
ko00511	Other glycan degradation	0.0686352275346951	0.162055398345808
ko00520	Amino sugar and nucleotide sugar metabolism	0.22228109159979	0.352849031711183

Continued on next page

Table S3 continued from previous page

var	description	p.value	adj.p.value
ko00521	Streptomycin biosynthesis	0.548190496230189	0.612247080140609
ko00523	Polyketide sugar unit biosynthesis	0.77183682571355	0.790435303441587
ko00531	Glycosaminoglycan degradation	0.111275682208652	0.222551364417304
ko00540	Lipopolysaccharide biosynthesis	0.309533323136244	0.417624324866361
ko00550	Peptidoglycan biosynthesis	0.281930058958137	0.409641966007549
ko00561	Glycerolipid metabolism	0.048646418063177	0.130395463460443
ko00562	Inositol phosphate metabolism	0.932085908031704	0.937601209262661
ko00564	Glycerophospholipid metabolism	0.177499244336365	0.298761104328536
ko00590	Arachidonic acid metabolism	0.529213341500229	0.595803099702244
ko00591	Linoleic acid metabolism	0.0435979064672159	0.127359054140066
ko00600	Sphingolipid metabolism	0.228341920646813	0.356129601008791
ko00601	Glycosphingolipid biosynthesis - lacto and neolacto series	0.378860113653472	0.48064342776933
ko00620	Pyruvate metabolism	0.158766476818118	0.281148969365417
ko00621	Dioxin degradation	0.0939893249850366	0.207508899317613
ko00622	Xylene degradation	0.170721183912626	0.296148992501494
ko00623	Toluene degradation	0.287992139841929	0.414903930280745
ko00624	Polycyclic aromatic hydrocarbon degradation	0.00173178197412691	0.0464880871102599
ko00625	Chloroalkane and chloroalkene degradation	0.015809807253893	0.0895889077720602
ko00626	Naphthalene degradation	0.632255082143037	0.675995999775574
ko00627	Aminobenzoate degradation	0.0479460664582554	0.130395463460443
ko00630	Glyoxylate and dicarboxylate metabolism	0.0627746570142626	0.15286668180863
ko00633	Nitrotoluene degradation	0.197928582237861	0.323537105581118
ko00640	Propanoate metabolism	0.0381721313534747	0.127240437844916
ko00643	Styrene degradation	0.0528641559022956	0.136165250051367
ko00650	Butanoate metabolism	0.0309073893101109	0.111792684738699
ko00660	C5-Branched dibasic acid metabolism	0.0528238385691674	0.136165250051367
ko00670	One carbon pool by folate	0.0327433200051196	0.115965925018132
ko00680	Methane metabolism	0.453903332044181	0.551168331767934
ko00710	Carbon fixation in photosynthetic organisms	0.0104148413727047	0.0686048857063766
ko00720	Carbon fixation pathways in prokaryotes	0.00404212560165608	0.0464880871102599
ko00730	Thiamine metabolism	0.237191738368523	0.36326662633017
ko00740	Riboflavin metabolism	0.0479907303302237	0.130395463460443
ko00750	Vitamin B6 metabolism	0.138732724857981	0.25635394810714
ko00760	Nicotinate and nicotinamide metabolism	0.0044560399556544	0.0464880871102599
ko00770	Pantothenate and CoA biosynthesis	0.0305364884943333	0.111792684738699
ko00780	Biotin metabolism	0.0428970379542919	0.127359054140066
ko00785	Lipoic acid metabolism	0.348879302577397	0.456226780293519
ko00790	Folate biosynthesis	0.00176493503495431	0.0464880871102599

Continued on next page

Table S3 continued from previous page

var	description	p.value	adj.p.value
ko00791	Atrazine degradation	0.0136610633961606	0.0829421706195466
ko00830	Retinol metabolism	0.13695911190839	0.255857681587102
ko00860	Porphyrin and chlorophyll metabolism	0.0660710750658963	0.158198348749329
ko00900	Terpenoid backbone biosynthesis	0.0998245645943331	0.209508345444897
ko00901	Indole alkaloid biosynthesis	0.529213341500229	0.595803099702244
ko00903	Limonene and pinene degradation	0.000975376876356915	0.0464880871102599
ko00906	Carotenoid biosynthesis	0.00328580346661821	0.0464880871102599
ko00908	Zeatin biosynthesis	0.495355908894636	0.578674299260585
ko00909	Sesquiterpenoid biosynthesis	0.819378125331846	0.834097492852777
ko00910	Nitrogen metabolism	0.0205151166232777	0.102575583116388
ko00920	Sulfur metabolism	0.00518847801131957	0.0464880871102599
ko00930	Caprolactam degradation	0.0257925354240788	0.109618275552335
ko00941	Flavonoid biosynthesis	0.00133326904903391	0.0464880871102599
ko00943	Isoflavonoid biosynthesis	0.106680988337322	0.221167902650545
ko00960	Tropane, piperidine and pyridine alkaloid biosynthesis	0.00161358286374227	0.0464880871102599
ko00965	Betalain biosynthesis	0.00504840454403284	0.0464880871102599
ko00970	Aminoacyl-tRNA biosynthesis	0.33990973535317	0.447943062093324
ko00980	Metabolism of xenobiotics by cytochrome P450	0.272885960356585	0.399919079832926
ko00983	Drug metabolism - other enzymes	0.0756733033967721	0.171526154366017
ko01040	Biosynthesis of unsaturated fatty acids	0.110953135071423	0.222551364417304
ko01051	Biosynthesis of ansamycins	0.0428665526276877	0.127359054140066
ko01053	Biosynthesis of siderophore group nonribosomal peptides	0.0830323744767739	0.185730311329626
ko01055	Biosynthesis of vancomycin group antibiotics	0.513855516785977	0.5942546792763
ko01056	Biosynthesis of type II polyketide backbone	0.61404969771612	0.664894577144844
ko01057	Biosynthesis of type II polyketide products	0.674488445692829	0.712192768743981
ko01501	beta-Lactam resistance	0.4074120626694	0.505547814991226
ko02010	ABC transporters	0.224162914263575	0.352849031711183
ko02020	Two-component system	0.415945774291851	0.512396968330541
ko02030	Bacterial chemotaxis	0.236513739484452	0.36326662633017
ko02040	Flagellar assembly	0.305586086985278	0.417624324866361
ko02060	Phosphotransferase system (PTS)	0.00215312125926874	0.0464880871102599
ko03008	Ribosome biogenesis in eukaryotes	0.0615204102652959	0.15286668180863
ko03010	Ribosome	0.493505101677743	0.578674299260585
ko03013	RNA transport	0.0292795765552897	0.111792684738699
ko03015	mRNA surveillance pathway	0.708957468508748	0.743967713867205
ko03018	RNA degradation	0.266530961692836	0.394002291198105

Continued on next page

Table S3 continued from previous page

var	description	p.value	adj.p.value
ko03020	RNA polymerase	0.0990619302785427	0.209508345444897
ko03022	Basal transcription factors	0.728314540150365	0.750384677730679
ko03030	DNA replication	0.581528673655639	0.637805642073926
ko03050	Proteasome	0.0231407726034991	0.10632246871878
ko03060	Protein export	0.168380533165437	0.295099903485817
ko03070	Bacterial secretion system	0.0538032101526983	0.13651560785013
ko03410	Base excision repair	0.312208812255998	0.417917307744249
ko03420	Nucleotide excision repair	0.483985706062325	0.575367622591575
ko03430	Mismatch repair	0.551022372126548	0.612247080140609
ko03440	Homologous recombination	0.573055579269966	0.632593821272041
ko03450	Non-homologous end-joining	0.00326898687368849	0.0464880871102599
ko04075	Plant hormone signal transduction	0.952893659200644	0.952893659200644
ko04080	Neuroactive ligand-receptor interaction	0.446377819495163	0.545929707296243
ko04112	Cell cycle - Caulobacter	0.0629451042741417	0.15286668180863
ko04113	Meiosis - yeast	0.00540506992343259	0.0464880871102599
ko04115	p53 signaling pathway	0.118492686013156	0.228906325252687
ko04122	Sulfur relay system	0.0100121370267844	0.0686048857063766
ko04141	Protein processing in endoplasmic reticulum	0.115305533030373	0.225727843923425
ko04142	Lysosome	0.0225348151364438	0.10632246871878
ko04144	Endocytosis	0.262799567959273	0.391894092570845
ko04146	Peroxisome	0.0104925119315635	0.0686048857063766
ko04210	Apoptosis	0.00308354591017269	0.0464880871102599
ko04310	Wnt signaling pathway	0.396185030658973	0.498899668237226
ko04614	Renin-angiotensin system	0.259753387164152	0.390779432016867
ko04621	NOD-like receptor signaling pathway	0.36085477929954	0.46828482809864
ko04626	Plant-pathogen interaction	0.481552881990763	0.575367622591575
ko04910	Insulin signaling pathway	0.215258218543312	0.345225444833614
ko04974	Protein digestion and absorption	0.0442010834956701	0.127359054140066
ko05010	Alzheimer's disease	0.529213341500229	0.595803099702244
ko05012	Parkinson's disease	0.899521077927273	0.910229662188312
ko05016	Huntington's disease	0.529213341500229	0.595803099702244
ko05100	Bacterial invasion of epithelial cells	0.133601587640573	0.255194043807835
ko05110	Vibrio cholerae infection	0.0282368821541232	0.111792684738699
ko05111	Vibrio cholerae pathogenic cycle	0.176944867970629	0.298761104328536
ko05120	Epithelial cell signaling in Helicobacter pylori infection	0.301438753114535	0.417624324866361
ko05130	Pathogenic Escherichia coli infection	0.378860113653472	0.48064342776933
ko05131	Shigellosis	0.13695911190839	0.255857681587102
ko05142	Chagas disease (American trypanosomiasis)	0.307806865640602	0.417624324866361
ko05143	African trypanosomiasis	0.115519543654929	0.225727843923425

Continued on next page

Table S3 continued from previous page

var	description	p.value	adj.p.value
ko05146	Amoebiasis	0.154990667338976	0.277351720501326
ko05150	Staphylococcus aureus infection	0.191746276282395	0.316474436582593
ko05322	Systemic lupus erythematosus	0.014284136981751	0.0837345960999197
ko05410	Hypertrophic cardiomyopathy (HCM)	0.188362101856462	0.313936836427437

The table lists relative pathway abundances and groupwise comparisons among NonBPD7, BPD7m, and BPD7s. No significant differences were observed in bile acid–related pathways, including primary and secondary bile acid biosynthesis (ko00120 and ko00121).

Table S4. Spearman correlations between differential bile acids and microbial genera.

Omics_1	Omics_2	Correlation	P-Value	FDR
CDCA-3S	g_Brevundimonas	-0.5000	2.18e-04	1.66e-03
CA	g_Brevundimonas	-0.6436	4.63e-07	5.72e-05
CA	g Burkholderia g	-0.5315	7.14e-05	8.16e-04
CA	Delftia	-0.5833	8.77e-06	1.75e-04
CA	g_Achromobacter	-0.5631	2.07e-05	3.68e-04
CA	g Ochrobactrum g	-0.5081	1.65e-04	1.55e-03
CDCA	Brevundimonas g	-0.6160	1.91e-06	7.33e-05
CDCA	Burkholderia g	-0.5031	1.96e-04	1.57e-03
CDCA	Delftia	-0.5280	8.12e-05	8.66e-04
CDCA	g_Achromobacter	-0.5384	5.51e-05	7.17e-04
CDCA	g Ochrobactrum g	-0.5036	1.93e-04	1.57e-03
HCA	Brevundimonas g	-0.6231	1.34e-06	7.17e-05
HCA	Burkholderia g	-0.5113	1.48e-04	1.48e-03
HCA	Delftia	-0.6354	7.14e-07	5.72e-05
HCA	g_Achromobacter	-0.5925	5.81e-06	1.55e-04
coproCA	g_Brevundimonas	-0.5369	5.83e-05	7.17e-04
coproCA	g_Delftia	-0.5520	3.25e-05	4.72e-04
7-KLCA	g_Brevundimonas	-0.5843	8.39e-06	1.75e-04
7-KDCA	g_Brevundimonas	-0.6124	2.29e-06	3.18e-03
7-KDCA	g_Delftia	-0.5043	1.88e-04	2.30e-03
HDCA	g_Brevundimonas	-0.5563	2.74e-05	4.38e-04

The table lists pairwise correlations (ρ), raw p-values, and false discovery rate (FDR)-adjusted p-values for associations between significantly altered bile acids and genera identified by LEfSe. Significant correlations were defined as $|\rho| > 0.5$ and FDR < 0.05.

Table S5. Performance comparison of machine learning models in five-fold cross-validation.

Model	Accuracy Mean	Accuracy Std	F1 Macro Mean	ROC AUC Mean
RandomForest	0.7747	0.0744	0.7773	0.9314
XGBoost	0.7593	0.0815	0.7576	0.9117
NaiveBayes	0.7901	0.0794	0.7743	0.8893
LogisticRegression	0.6209	0.0850	0.6011	0.8526
KNN	0.6352	0.0794	0.6311	0.7906
SVM	0.4857	0.1447	0.4463	0.7661

The table summarizes evaluation metrics (macro-F1, accuracy, precision, recall, and AUC) for six classifiers—random forest (RF), extreme gradient boosting (XGBoost), logistic regression (LR), naive Bayes (NB), k-nearest neighbor (KNN), and support vector machine (SVM). Tree-based models (RF and XGBoost) achieved the best predictive performance, with AUCs of 0.93 and 0.91, respectively.

1.2 Figures

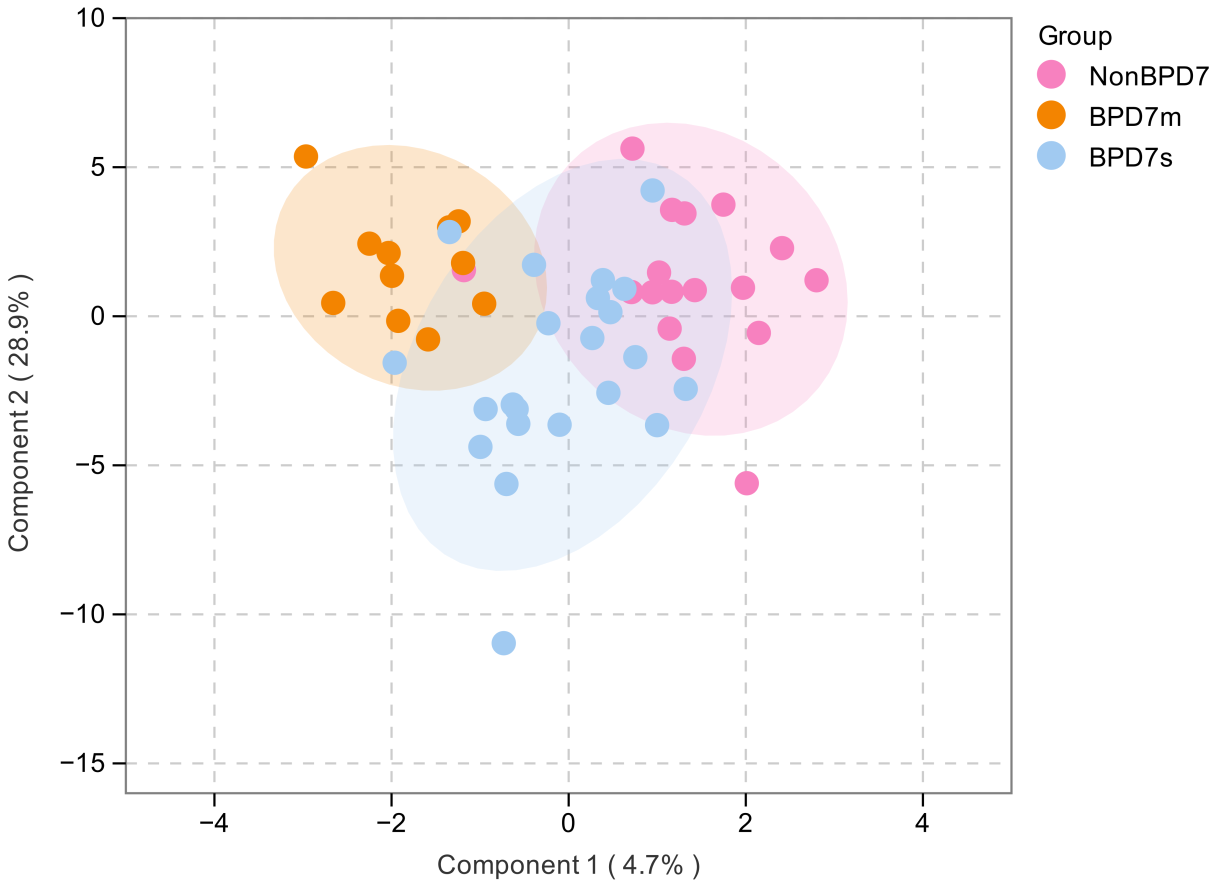

Figure S1: OPLS-DA score plot showing separation among NonBPD7, BPD7m, and BPD7s groups (Component 1: 4.7%; Component 2: 28.9%).

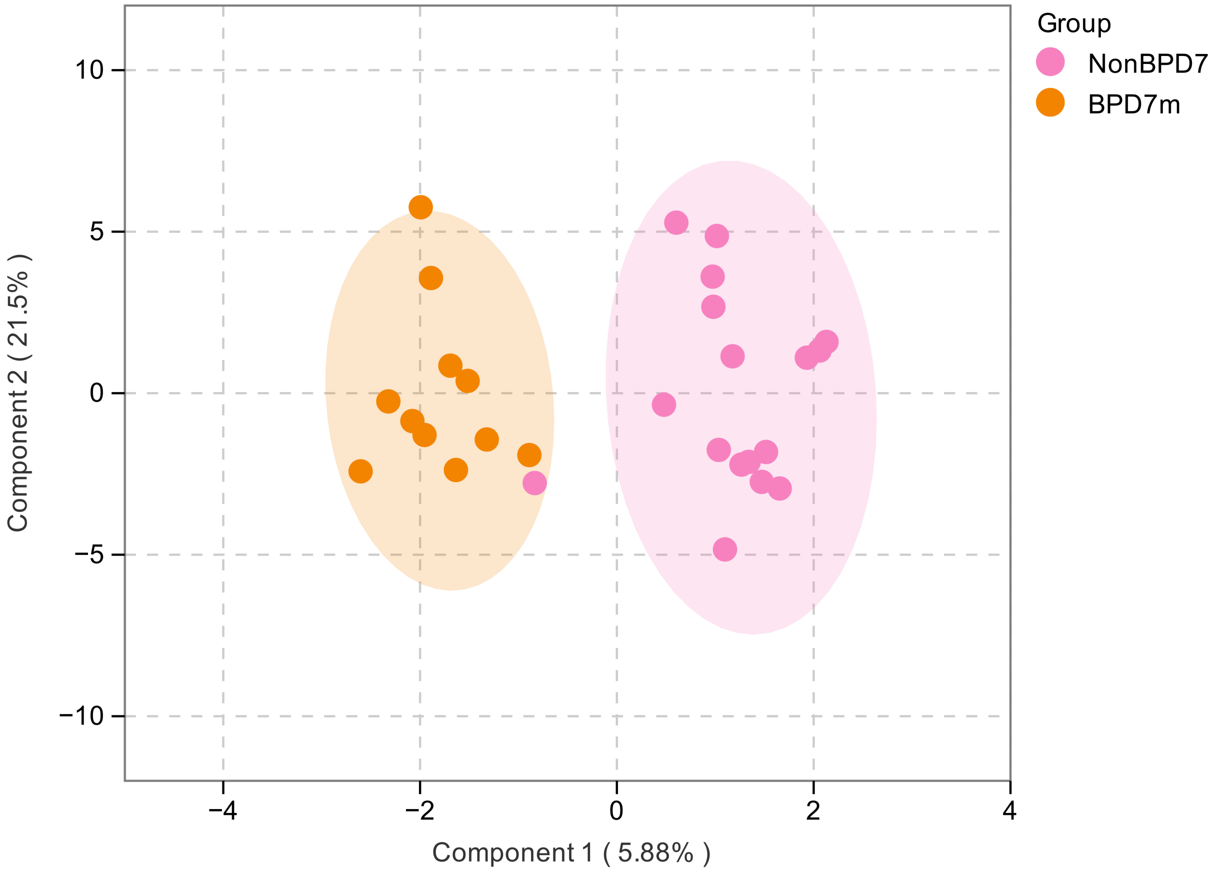

Figure S2: OPLS-DA score plot showing discrimination between NonBPD7 and BPD7m groups (Component 1: 5.9%; Component 2: 21.5%).

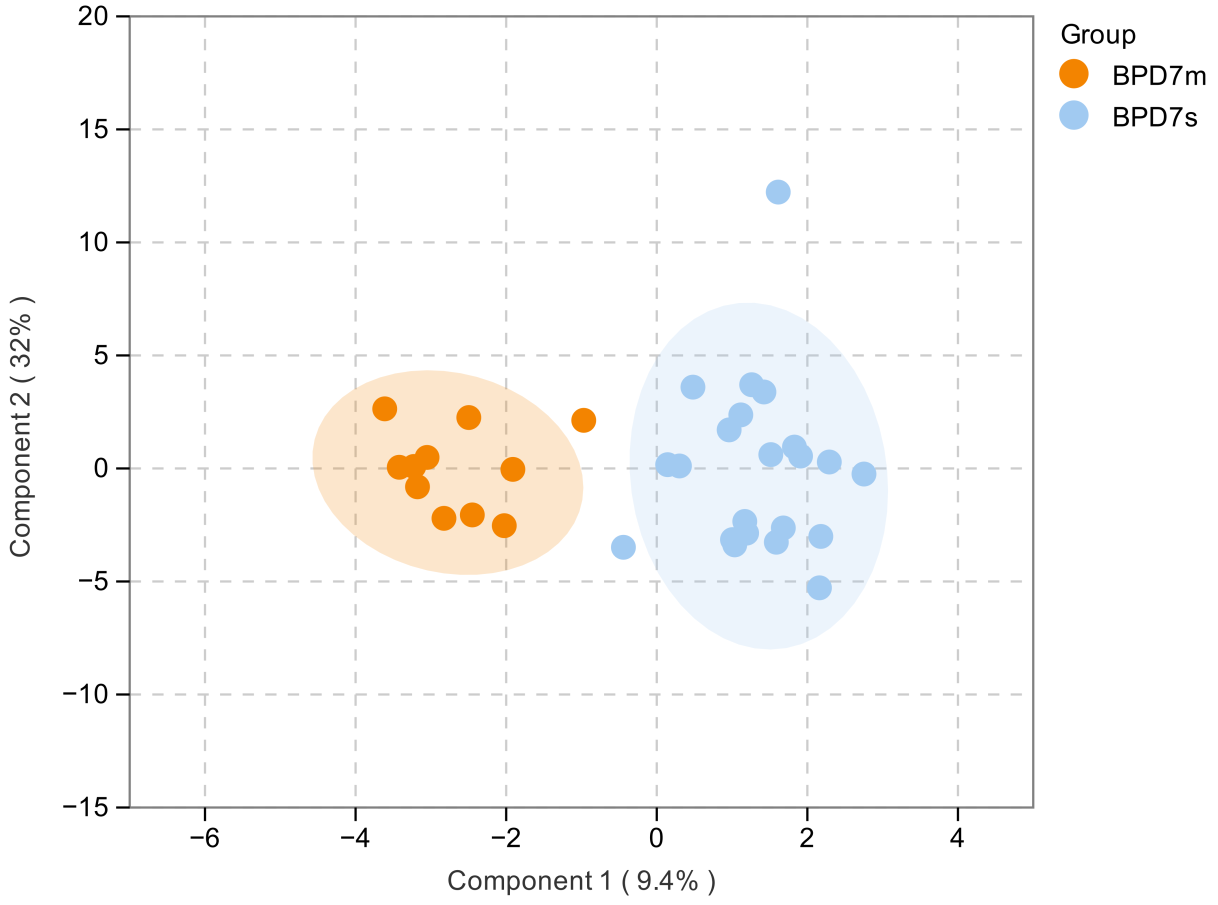

Figure S3: OPLS-DA score plot showing discrimination between BPD7m and BPD7s groups (Component 1: 9.4%; Component 2: 32.0%).

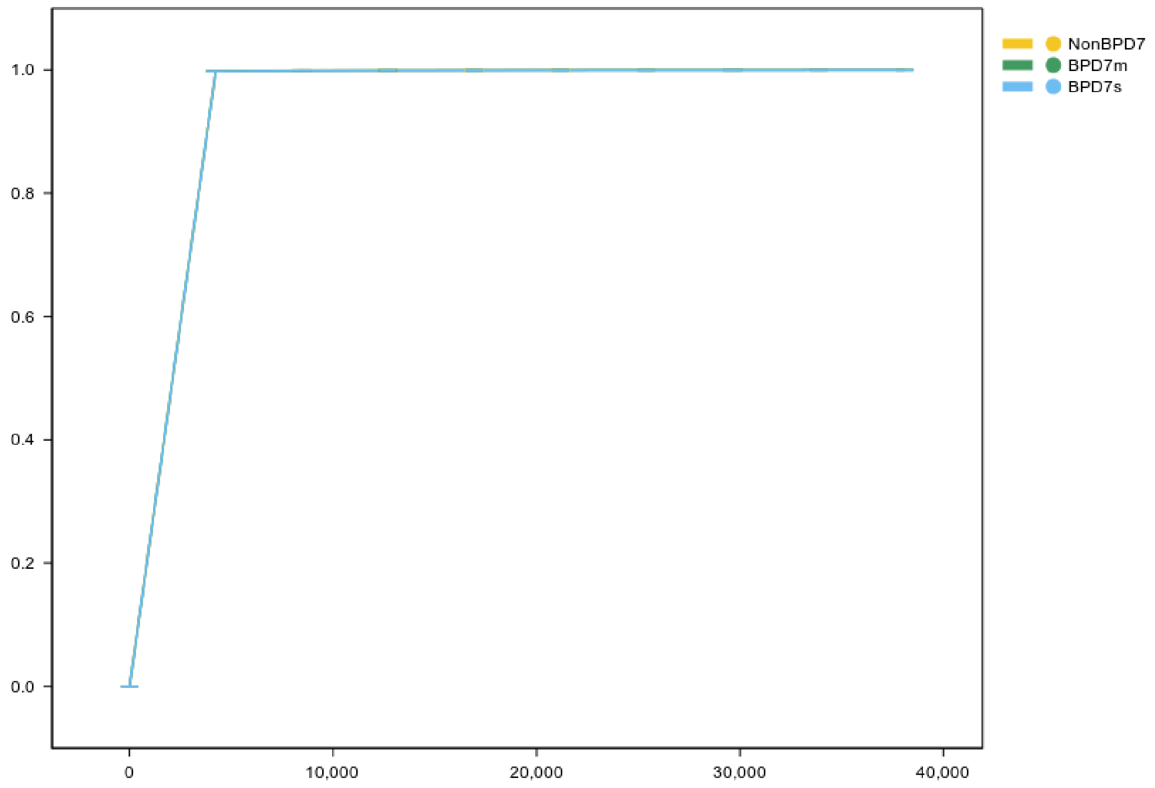

Figure S4: Rarefaction curves and Good's coverage. Rarefaction curves plateaued in all groups (NonBPD7, BPD7m, BPD7s), and Good's coverage exceeded 0.99, indicating sufficient sequencing depth.

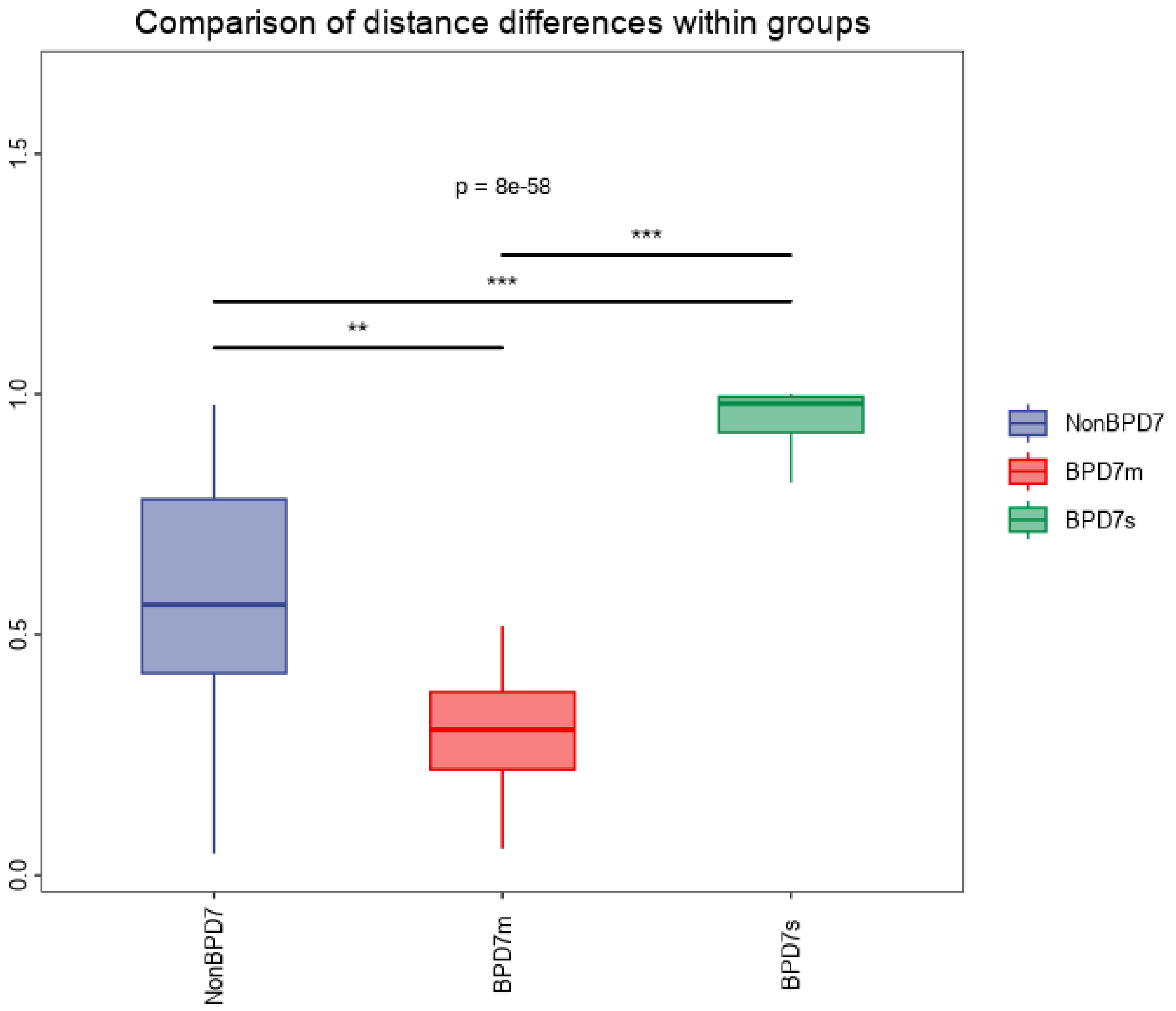

Figure S5: β-diversity analysis based on Bray–Curtis distances. Boxplots show distribution of pairwise distances among NonBPD7, BPD7m, and BPD7s. According to PERMANOVA (Supplementary Table S2), groups were significantly separated (Pseudo-$F = 3.0137$, $R^2 = 0.1137$, $p = 0.001$). Dispersion tests indicated significant within-group differences.

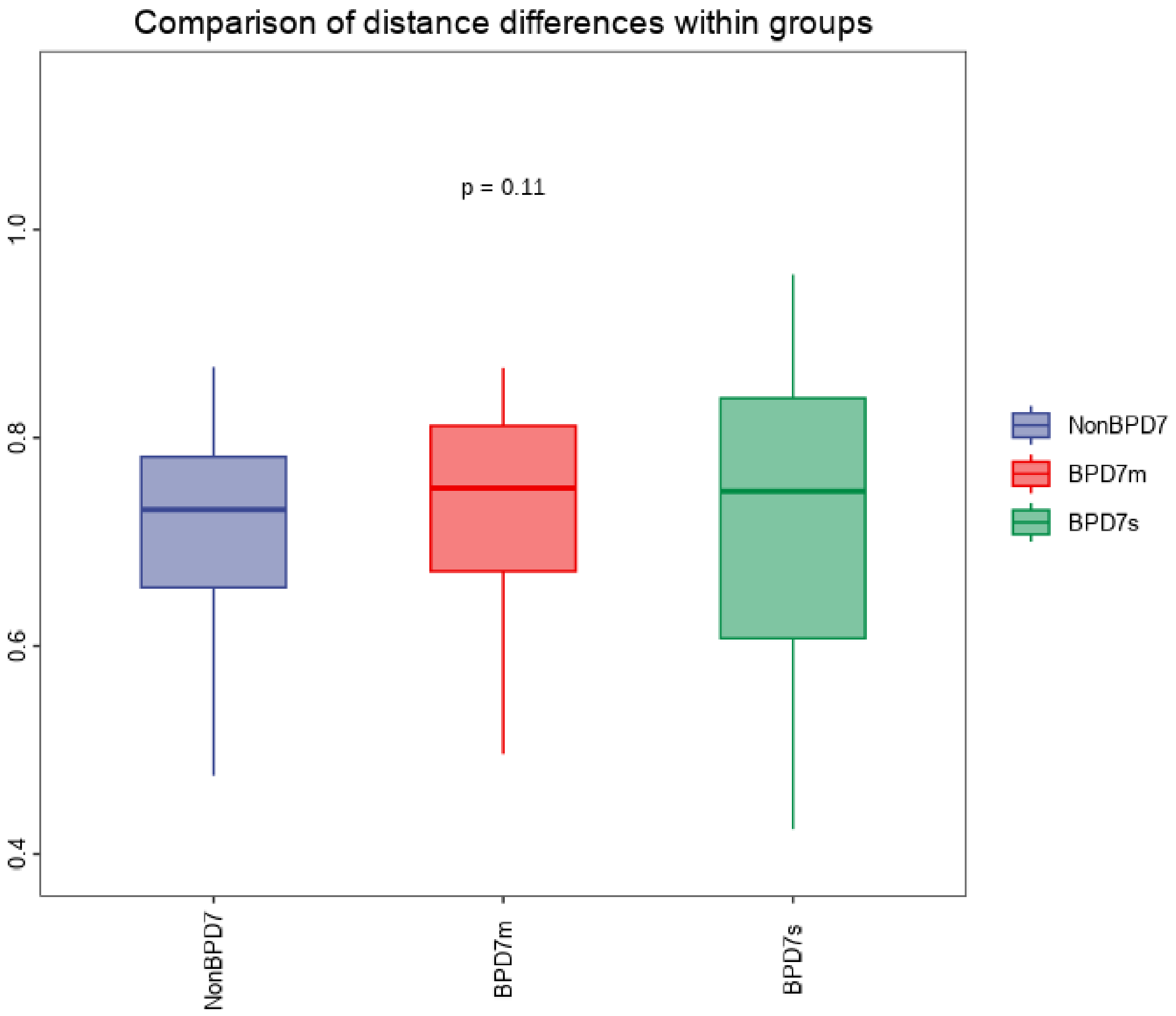

Figure S6: β-diversity analysis based on unweighted UniFrac distances. Boxplots show distribution of pairwise distances among NonBPD7, BPD7m, and BPD7s. PERMANOVA (Supplementary Table S2) indicated weak but significant separation (Pseudo-$F = 1.3704$, $R^2 = 0.0551$, $p = 0.041$). Dispersion tests showed no significant within-group differences.

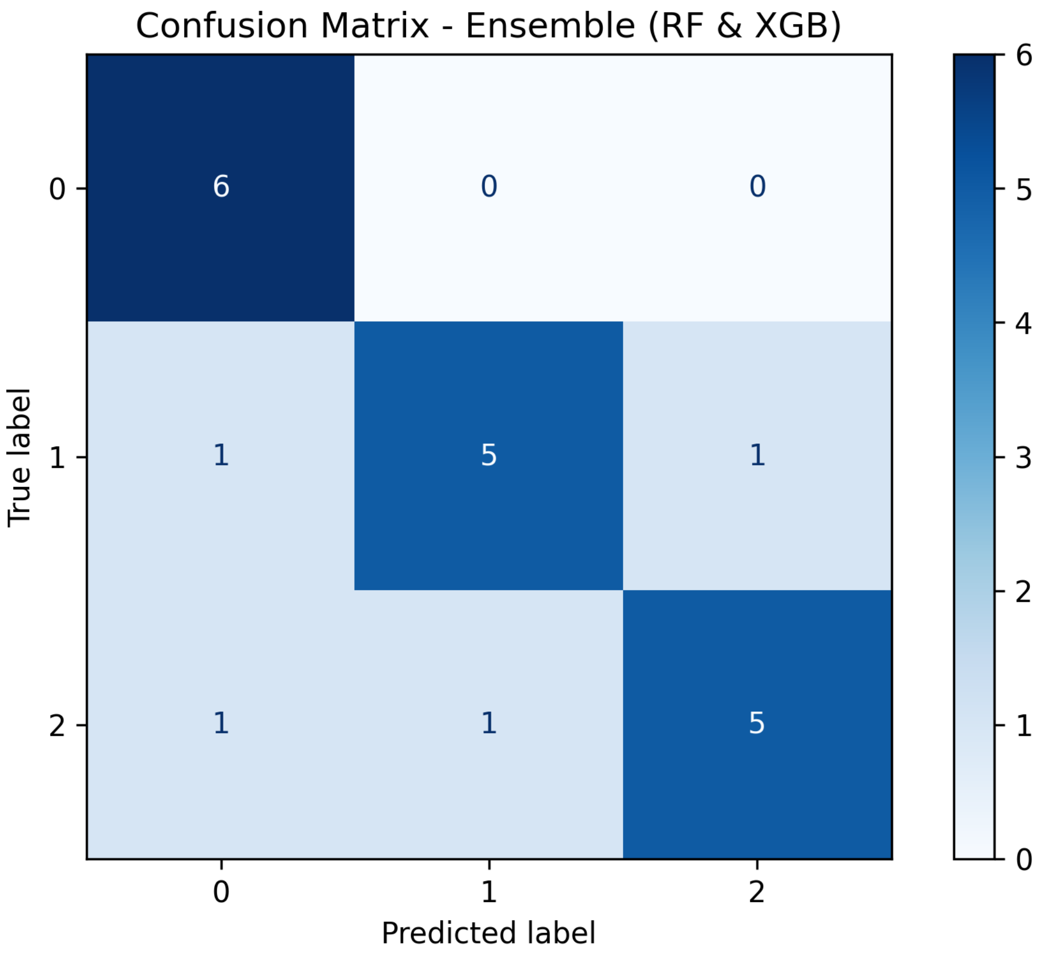

Figure S7: Confusion matrix of the ensemble model (Random Forest + XGBoost) on the independent test set. Rows indicate true labels and columns predicted labels (0 = NonBPD7, 1 = BPD7m, 2 = BPD7s).

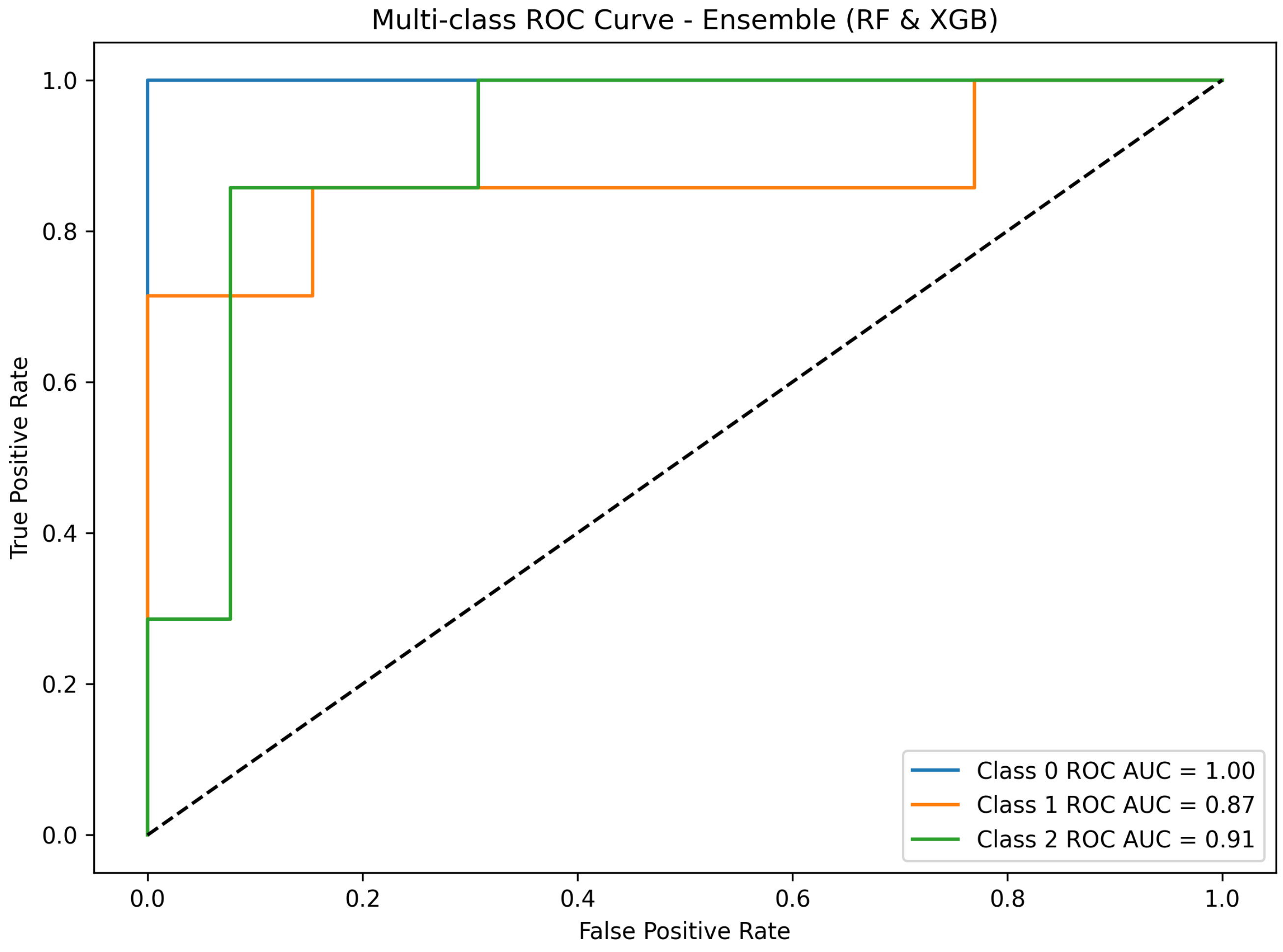

Figure S8: Multi-class ROC curves of the ensemble model (Random Forest + XGBoost) showing class-specific AUCs.

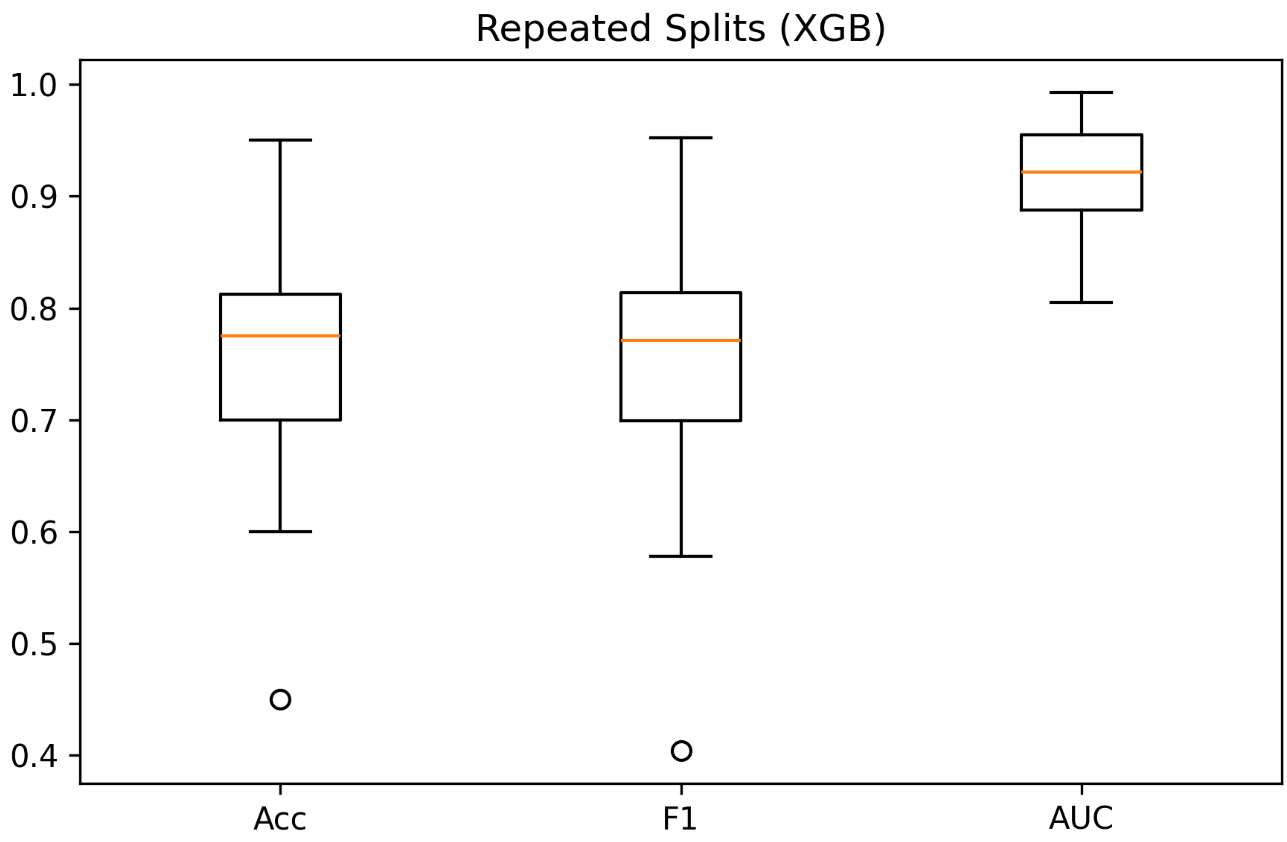

Figure S9: Performance stability of XGBoost under repeated stratified splits. Boxplots summarize accuracy, F1, and AUC distributions across multiple runs.

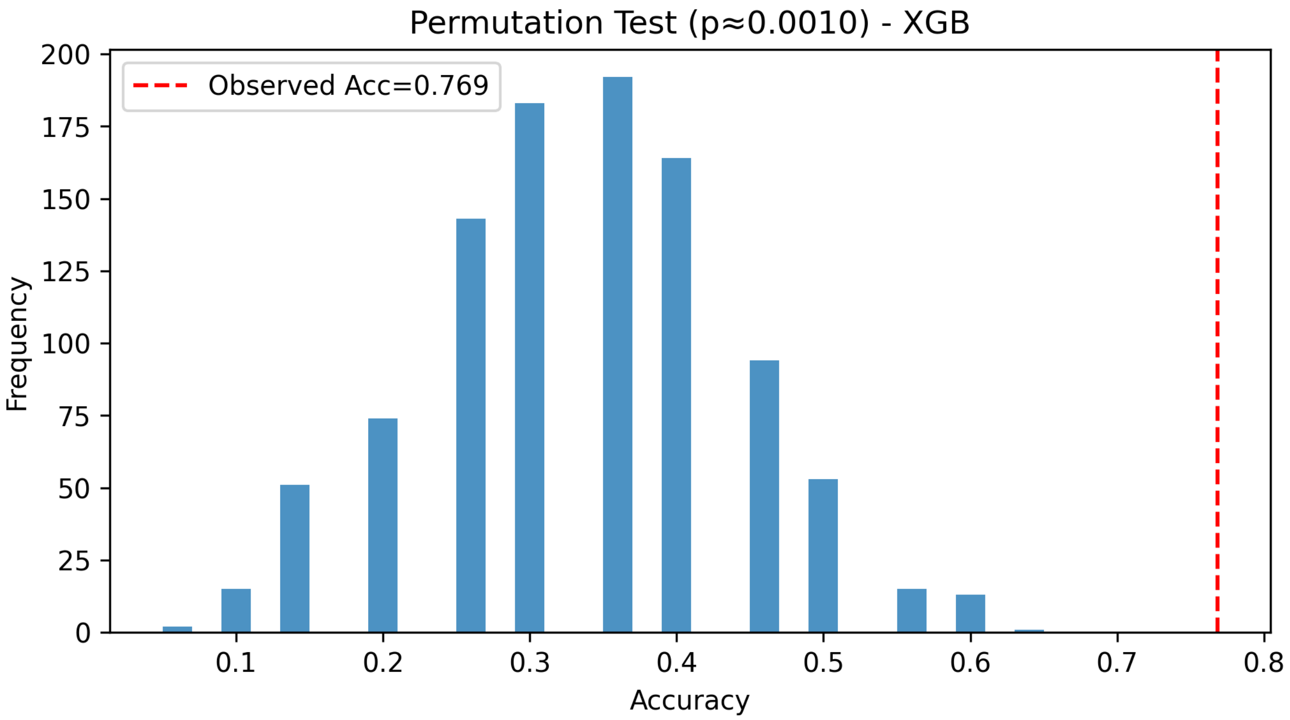

Figure S10: Permutation test for the XGBoost classifier. Histogram shows the distribution of accuracy under label shuffling; the red dashed line indicates the observed accuracy.

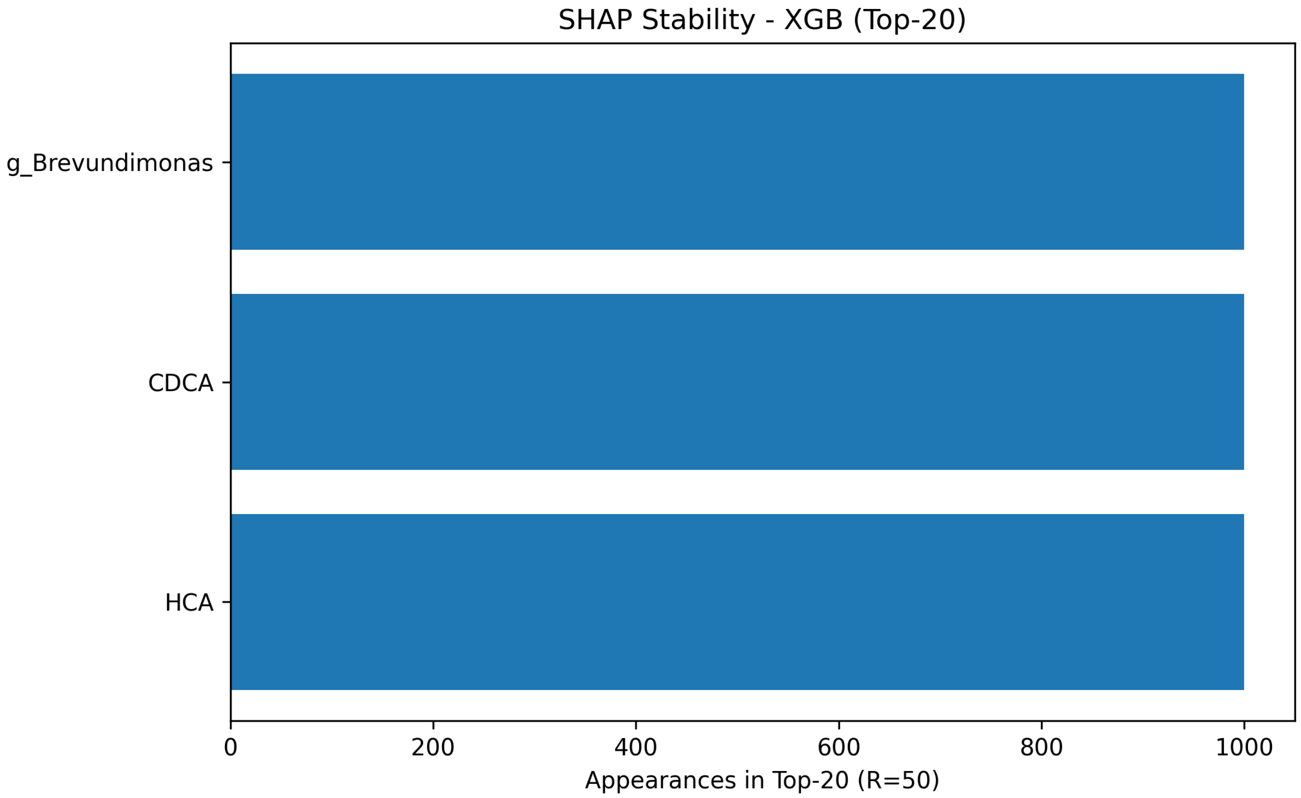

Figure S11: SHAP stability analysis of the XGBoost classifier. The analysis considered the top-20 ranked features across 50 resampling runs. Only three features (*Brevundimonas*, CDCA, HCA) consistently appeared in the top-20 in all runs and are therefore shown.
